# Long-term prognosis of adults with moderately severe SARS-CoV-2 lower respiratory tract infection managed in primary care: Prospective cohort study

**DOI:** 10.1080/13814788.2025.2501306

**Published:** 2025-06-02

**Authors:** Tamara N. Platteel, Johannes C. Koelmans, Daniela Cianci, Natasha J. H. Broers, Eefje G. P. M. de Bont, Jochen W. L. Cals, Roderick P. Venekamp, Theo J. M. Verheij

**Affiliations:** ^a^ Department of General Practice & Nursing Science, Julius Center for Health Sciences and Primary Care, University Medical Center Utrecht, Utrecht University, Utrecht, The Netherlands; ^b^ Department of Family Medicine, Care and Public Health Research Institute (CAPHRI), Maastricht University, Maastricht, The Netherlands

**Keywords:** COVID-19, primary care, long-term prognosis

## Abstract

**Background:**

Information about the incidence of persisting symptoms after COVID-19 and its impact on patients treated in primary care are scarce.

**Objectives:**

To determine differences in health-related quality of life (HRQoL) and symptomatology between adults with and without SARS-CoV-2 moderately severe lower respiratory tract infection (LRTI) in the 12 months following their primary care visit.

**Methods:**

Prospective cohort study in 35 Dutch practices. Individuals aged ≥18 years who presented to their general practitioner (GP) with a moderately severe LRTI during the first COVID-19 waive in The Netherlands (March–June 2020, *n* = 277; 268 (97%) with complete follow-up) were included between September and December 2020, then underwent serology testing (participants, GPs and study personnel remained blinded for serology outcomes during study conduct) and completed baseline and follow-up questionnaires. Main outcome measures: (1) SF-36 scores, and (2) risk of persisting symptoms during 12 months follow-up.

**Results:**

The change in SF-36 PSC (*p* = 0.13), MCS (*p* = 0.30) during 12 months follow-up did not differ between SARS-CoV-2 serology positive and negative participants after adjusting for sex, age, BMI, diabetes, and chronic pulmonary conditions. The risk of any persisting symptom during 12 months follow-up did not significantly differ between the groups (aHR 0.61, 95% CI 0.33–1.15), nor did the risk of individual symptoms.

**Conclusions:**

In the 12 months following their moderately severe LRTI, primary care patients with and without confirmed SARS-CoV-2 infection had a comparable HRQoL profile. A substantial proportion of participants reported persisting symptoms, indicating that persisting symptoms can occur following LRTIs irrespective of causative pathogen.

## Introduction

COVID-19, caused by SARS-CoV-2, has led to a global crisis. During the early stages of the pandemic, hospital and ICU admissions and mortality were the main focus. However, COVID-19’s impact extends beyond hospitals, with many non-hospitalised patients experiencing persistent symptoms like fatigue, dyspnoea, chest pain, anosmia, mental health issues, and cognitive deficits [1,2]. This condition, lasting over 4 weeks, is known as post-acute COVID-19 or long-COVID [1].

Although evidence on long-COVID is accumulating rapidly, information about its incidence and impact in patients treated in primary care is scarce. The few studies available report long-COVID symptoms in 28%–64% of patients 4 weeks to 12 months after onset [3–8]. These figures are, however, difficult to interpret due to the absence of an adequate control group. This is an important omission because lower respiratory tract infections (LRTIs) caused by other viral and bacterial pathogens can also lead to long-lasting sequelae. Additionally, perceived symptoms might be influenced by COVID-19 restrictions and awareness of having this new viral infection with unknown disease course [9].

A few studies tried to mitigate this risk of bias by including a SARS-CoV-2 negative control group [10–14]. However, to truly prevent bias, a double-blinded study design is warranted. During the first wave of COVID-19 (March–May 2020) in the Netherlands, the lack of SARS-CoV-2 tests for primary care LRTI patients allowed us to conduct such a study.

## Methods

We prospectively followed patients presenting to primary care with a moderately severe LRTI for 12 months and determined differences in health-related quality of life (HRQoL) and presence and duration of long-COVID symptoms between those with and without established SARS-CoV-2 infection based on serology testing at moment of inclusion (i.e. 4–11 months post-index consultation), while keeping participants, general practitioners (GPs) and study personnel blinded for serology outcomes. The study was registered with the Dutch Trial Register (NTR) number NL8729.

### Setting and study population

Adult patients presenting to primary care between March 1st and June 1st 2020 with moderately severe LRTI (defined as International Classification of Primary Care (ICPC) code R81 (pneumonia) or codes R02 (dyspnoea), R05 (cough), R74 (respiratory tract infection), R78 (bronchitis), or R83 (other respiratory tract infection) for which the GP prescribed oral antibiotics) were retrospectively selected (between September and November 2020) from 35 GP practices in Noord-Brabant and Utrecht, the Netherlands. At the time of index consultation, widescale COVID-19 PCR tests were not available in The Netherlands, which limited testing to hospitalised patients and health care workers. Patients admitted to the hospital 14 days before or after the index consultation (i.e. patients’ first primary care contact for the LRTI episode), those with a life expectancy of less than a year and those unable to complete questionnaires were excluded from participation.

Potentially eligible participants were informed about the study by their GP, and those interested received verbal and written information from the study team. Written informed consent was obtained from all participants after selection between September and November 2020.

### Study procedures, baseline and follow-up measurements

At baseline, defined as the moment of inclusion in the study (September–December 2020), participants provided a venous blood sample for Abbott Alinity SARS-CoV-2 spike-specific quantitative IgG serology testing, which remains detectable for up to seven months after symptom onset in at least 92% of patients (13). Participants, GPs and study personnel remained blinded for the serology test results until follow-up and analyses were completed.

Participants completed a questionnaire about demographics and symptoms (cough, dyspnoea, chest pain, fatigue, brain fog, headache, anosmia/ageusia) using a 0–4 Likert scale (0: absent, 1: a little, 2: moderate, 3: much and 4: very much) at time of the index consultation (recall) and at baseline. They also assessed their HRQoL two weeks prior to the index consultation (recall) and at baseline using the SF-36 questionnaire [15]. The SF-36 questionnaire consists of a single item of health transition (HT) and a further 35 items which can be divided into eight subscales: (1) physical function (PF), (2) limitations due to physical health problems (role physical, RP), (3) bodily pain (BP), (4) general health (GH), (5) vitality (VT), (6) social functioning (SF), (7) limitations due to emotional health problems (RE), and (8) mental health (MH). Scores range from 0 to 100, with higher scores indicating better functional status. The eight subdomain scores were aggregated into two summary measures: physical component summary (PCS) and mental component summary (MCS) scores.

**Figure 1.**
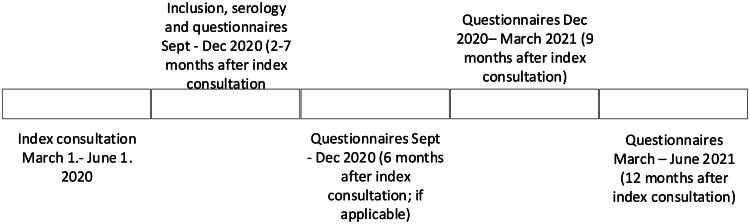
Timeline index consultation, baseline and follow-up measurements.

Follow-up HRQoL and symptom questionnaires were performed every 3 months up to 12 months after the index consultation. Participants included more than six months after the baseline consultation completed questionnaires only at inclusion, 9 months, and 12 months post-inclusion (Figure 1). Participants also reported any positive SARS-COV-2 test results during follow-up.

Data on signs and symptoms at the index consultation, comorbidities, medication use, and results of SARS-CoV-2 reverse-transcriptase polymerase chain reaction (RT-PCR) and serology tests were extracted from electronic primary care health records.

### Outcome measures

The primary outcome was the change in participants’ SF-36 physical component summary (PCS) scores during 12 months follow-up. Secondary outcomes included changes in SF-36 mental component summary (MCS) scores and the eight individual SF-36 subscales, as well as the time to resolution of symptoms.

### Sample size considerations

Assuming a mean SF-36 PCS score of 50 with a standard deviation (SD) of 10 and that at least 20% of moderately severe LRTI cases were caused by SARS-CoV-2, a sample size of 274 patients was required to achieve 80% power to detect a clinically significant difference of three or more points in the SF-36 PCS score between groups [16,17].

### Statistical analyses

Baseline characteristics were compared between those with and without established SARS-CoV-2 infection (based on serology testing) using appropriate statistical tests.

For the primary analysis, a linear mixed effects model was used with SF-36 PSC scores as the dependent variable and SARS-CoV-2 serology test result, time, age, gender, body mass index (BMI), diabetes, chronic pulmonary diseases, and the interaction term between time and SARS-CoV-2 serology test result as fixed effects. A random intercept and slope for time accounted for repeated measurements. The difference in change of SF-36 PSC score from 2 weeks prior to 12 months after the index consultation between serology-positive and negative groups was estimated with 95% confidence intervals (CIs). Differences in changes in SF-36 MCS scores and the eight individual SF-36 subscales between groups were estimated similarly.

For time to symptom resolution, Kaplan–Meier survival analyses with log-rank tests were performed. Participants with persisting symptoms were censored at the end of follow-up. Unadjusted hazard ratios (HRs) with 95% CIs were calculated using Cox proportional hazard modelling, adjusting for age, gender, and comorbidities (chronic pulmonary disease, cardiovascular disease, and diabetes). The proportional hazards assumption was tested using the supremum test, adding time-dependent coefficients when violated. Effect modification by chronic pulmonary disease, cardiovascular disease, and diabetes was assessed by adding interaction terms with SARS-CoV-2 serology test result.

Several assumptions were made: the study SARS-CoV-2 serology test results were used to attribute moderately severe LRTI at index consultation to SARS-CoV-2 infection, excluding participants with discrepancies between study and routine care RT-PCR or serology tests. Participants testing RT-PCR positive between one month after the index consultation and 14 days before the study serology were considered SARS-CoV-2 negative until the positive RT-PCR result. Participants with a negative serology test who experienced a SARS-CoV-2 infection during follow-up were censored from the moment of the positive test.

Post-hoc sensitivity analyses included (1) excluding participants who knew their SARS-CoV-2 status before the first questionnaire (deblinded participants), (2) excluding those with chronic pulmonary disease to avoid overestimating persisting symptoms in the SARS-CoV-2 negative group, and (3) defining symptom resolution as a Likert scale score ≤2 in the symptom duration analysis.

Data were analysed using IBM SPSS Statistics version 26.0.0.1 and SAS version 9.4 for the linear mixed effects model and the proportional hazards checks. Statistical significance was assumed at *p* < 0.05.

## Results

### Study population

**Figure 2.**
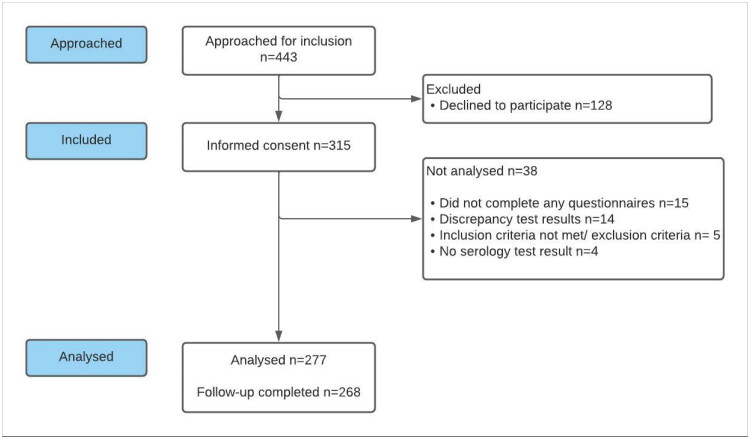
Patient selection flowchart.

Out of 443 patients with moderately severe LRTI approached for participation, 315 consented and 277 (88%) were suitable for analysis. Complete follow-up data was obtained for 97% of these 277 patients (Figure 2). The first follow-up questionnaire was completed after a median of 7.8 months (range 4.8–11.2; IQR 7.1–8.4) post-index consultation. Serology testing was performed after a median of 7.6 months (range 4.7–11.2; IQR 6.9–8.4) post-index consultation.

### Baseline characteristics

The SARS-CoV-2 serology test was positive in 19.9% (55/277) of participants (Table 1). Participants with a positive serology test were older (mean age 59.9 years [SD 10.7] vs 54.8 years [SD 14.6]) and more likely to have diabetes (20.0% vs 7.2%). Active smoking (17.1% vs 1.8%) and chronic pulmonary conditions (27.0% vs 10.9%) were more prevalent in SARS-CoV-2 serology-negative participants.

**Table 1.** Baseline characteristics, including demographics and clinical presentation of SARS-CoV-2 positive and negative participants.

Baseline variable	SARS-CoV-2 positive (*n* = 55)	SARS-CoV-2 negative (*n* = 222)	*p* value
**Age at index consultation, years**	59.9 ± 10.7	54.8 ± 14.6	<0.01
**Female sex**	61.8 (*n* = 34)	67.1 (*n* = 149)	0.53
**BMI, kg/m^2^**	28.7 ± 5.2	27.3 ± 5.6	0.09
**Comorbities**			
Diabetes	20.0 (*n* = 11)	7.2 (*n* = 16)	0.01
Cardiovascular disease	41.8 (*n* = 23)	39.2 (*n* = 87)	0.76
Chronic pulmonary disease	10.9 (*n* = 6)	27.0 (*n* = 49)	0.06
**Chronic medication at index consultation**			
Antihypertensive drugs	27.3 (*n* = 15)	25.2 (*n* = 56)	0.76
Anticoagulants/antiplatelet therapy	25.5 (*n* = 14)	19.8 (*n* = 44)	0.36
Inhalation medication	10.9 (*n* = 6)	23.4 (*n* = 52)	0.04
Psychotropic medication	18.2 (*n* = 10)	19.4 (*n* = 43)	0.84
**Smoking**			
Yes	1.8 (*n* = 1)	17.1 (*n* = 38)	<0.01
No	87.3 (*n* = 48)	73.9 (*n* = 164)	0.04
Former	10.9 (*n* = 6)	9.0 (*n* = 20)	0.67
**Living single (vs living with household members)**	23.6 (*n* = 13)	13.5 (*n* = 30)	0.09
**Educational level**			
Lower	29.1 (*n* = 16)	27.0 (*n* = 60)	0.76
Intermediate	43.6 (*n* = 24)	42.3 (*n* = 94)	0.77
Higher	27.3 (*n* = 15)	30.6 (*n* = 68)	0.63
**Ethnicity: Western European**	96.4 (*n* = 53)	93.7 (*n* = 208)	0.75
**Occupation**			
Employed	54.5 (*n* = 30)	55.9 (*n* = 124)	0.59
Unemployed	43.6 (*n* = 24)	41.4 (*n* = 92)	0.77
Student	1.8 (*n* = 1)	2.7 (*n* = 6)	0.71
**Premorbid scores SF-36**			
PCS	50.2 ± 8.6	47.3 ± 10.7	0.06
MCS	53.1 ± 8.7	52.4 ± 8.8	0.60
**Reported at index consultation**			
**Duration of symptoms**			
1–4 days	9.3 (*n* = 5)	17.2 (*n* = 35)	0.21
5–9 days	44.4 (*n* = 24)	29.6 (*n* = 60)	0.02
>9 days	46.3 (*n* = 25)	53.2 (*n* = 108)	0.67
**Symptoms**			
Cough	98.2 (*n* = 54)	96.4 (*n* = 214)	0.50
Fever	94.5 (*n* = 52)	74.8 (*n* = 166)	<0.01
Shortness of breath/dyspnoea	87.3 (*n* = 48)	91.4 (*n* = 203)	0.34
Sputum	69.1 (*n* = 38)	76.1 (*n* = 169)	0.28
Nasal congestion	20.0 (*n* = 11)	22.5 (*n* = 50)	0.69
Sore throat	10.9 (*n* = 6)	14.0 (*n* = 31)	0.55
Hemoptysis	9.1 (*n* = 5)	12.2 (*n* = 27)	0.52
Fatigue	100 (*n* = 55)	92.8 (*n* = 206)	0.04
Myalgia/arthralgia	89.1 (*n* = 49)	76.7 (*n* = 170)	0.41
Chills	78.2 (*n* = 43)	65.3 (*n* = 145)	0.07
Chest pain	74.5 (*n* = 41)	74.3 (*n* = 165)	0.97
Headache	72.7 (*n* = 40)	76.6 (*n* = 170)	0.55
Anosmia/ageusia	69.1 (*n* = 38)	41.4 (*n* = 92)	<0.01

The values are displayed as a percentage (*n*) or as mean ± standard deviation. Cardiovascular disease: Hypertension, Ischaemic heart disease, CVA/TIA, Congestive heart failure, Atrial fibrillation, Thrombosis, Peripheral artery disease. Chronic pulmonary disease: Asthma/COPD. BMI: Body mass index.

There were no statistically significant differences in baseline SF-36 PCS and MCS scores between SARS-CoV-2 serology-negative and positive participants (50.2 [SD 8.6] vs 47.3 [SD 10.7] and 53.1 [SD 8.7] vs 52.4 [SD8.8], respectively). Symptoms more prevalent in SARS-CoV-2 positive participants at index consultation included fever (94.5% vs 74.8%), myalgia/arthralgia (89.1% vs 76.6%), fatigue (100% vs 92.8%), and anosmia/ageusia (69.1% vs 41.4%).

### HRQoL scores during 12 months follow-up

**Figure 3.**
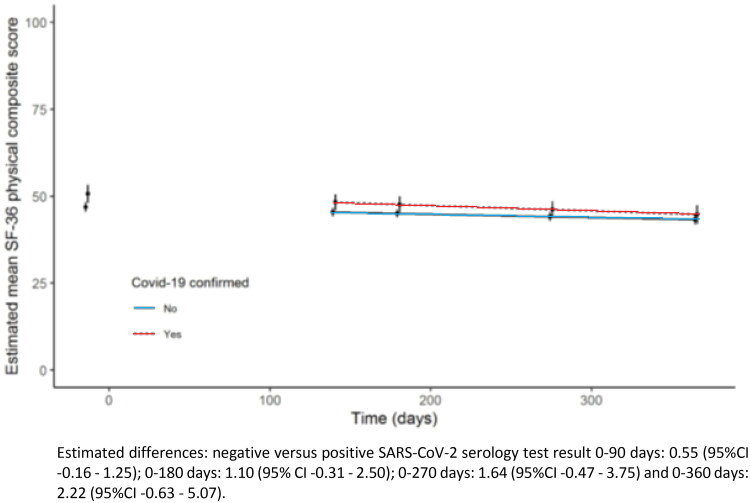
Estimated mean physical component summary (PCS) score (SF-36) over time, based on mixed model. Estimated differences: negative versus positive SARS-CoV-2 serology test result 0–90 days: 0.55 (95%CI −0.16 to 1.25); 0–180 days: 1.10 (95% CI −0.31 to 2.50); 0–270 days: 1.64 (95%CI −0.47 to 3.75) and 0–360 days: 2.22 (95%CI −0.63 to 5.07).

The change in SF-36 PSC score during 12 months follow-up did not differ statistically or clinically between SARS-CoV-2 serology-positive and negative participants (*p* = 0.127) after adjusting for sex, age, BMI, diabetes and chronic pulmonary conditions (Figure 3).

Similarly, there was no significant difference in the change in SF-36 MCS scores during 12 months follow-up (*p* = .302; Appendix S1), nor in the change in the eight individual SF-36 subscales (data not shown). Sensitivity analyses, censoring participants from the moment of deblending, yielded similar results.

### Time to resolution of symptoms

At least one persisting symptom 4 weeks after the index consultation was reported in 80% of SARS-CoV-2 serology-positive participants and in 83.8% of serology-negative participants. The most commonly reported persisting symptoms were dyspnoea (serology-positive vs. negative group: 54.5% vs 58.1%), fatigue (63.6% vs 68.0%), cough (45.5% vs 46.8%) and brain fog (43.6% vs 37.8%; Appendix S2).

**Figure 4.**
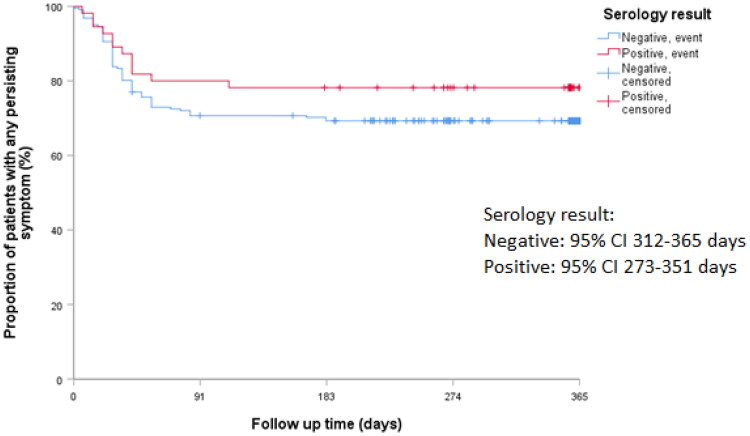
Kaplan–Meier curve of the proportion of patients with any persisting symptom over the 12 month follow up.

Kaplan–Meier survival curves for any persisting symptom over 12 months were comparable between the two groups with no statistical significant differences in log-rank tests (Figure 4, Table 2). The same was true for individual symptoms (cough, dyspnoea, chest pain, fatigue, brain fog, headache, and anosmia/ageusia; Appendix S3). The risk of any persisting symptom did not differ significantly between the groups in both unadjusted and adjusted Cox models (aHR 0.61, 95% CI 0.33–1.15; Table 2). No statistically significant hazard ratios were found for individual symptoms either (Table 2). There was no evidence of effect modification between the SARS-CoV-2 serology status and diabetes, chronic pulmonary disease and cardiovascular disease.

**Table 2.** Adjusted hazard ratio for persisting symptoms until 12 months follow-up for SARS-CoV-2 serology positive versus negative participants.

Symptom	P Log rank	Unadjusted Hazard ratio	95% CI	Adjusted Hazard ratio	95% CI
Dyspnoea	0.91	1.08	0.74–1.59	0.95	0.64–1.42
Cough	0.67	1.02	0.73–1.43	0.92	0.65–1.30
Fatigue	0.72	1.08	0.71–1.63	1.01	0.66–1.55
Brain fog	0.49	0.90	0.63–1.27	0.85	0.59–1.21
Anosmia/ageusia	0.43	0.78	0.57–1.08	0.78	0.56–1.09
Headache	0.07	1.13	0.82–1.55	1.01	0.73–1.41
Chest pain	0.83	1.03	0.75–1.43	0.96	0.69–1.34
Any symptom	0.21	0.68	0.37–1.26	0.61	0.33–1.15

This table provides statistical details to support the (visual) interpretation of the Kaplan–Meier curves (see Figure 3 for the Kaplan–Meier curve for any symptom and Appendix S3 for individual symptoms).

Sensitivity analyses, including (1) censoring participants from the moment they knew their COVID-19 status, (2) excluding participants with chronic pulmonary disease and (3) assuming symptom resolution for all participants with a Likert scale symptom score ≤2, also showed no statistically significant differences between the two groups in Cox proportional hazard models.

## Discussion

This is, to our knowledge, the first blinded study showing that over 12 months following moderately severe LRTI, primary care patients with and without confirmed SARS-CoV-2 infection had comparable HRQoL profiles. Despite a significant proportion of patients reporting persistent symptoms, no difference in symptom resolution during 12 months follow-up was found between the two groups.

Few methodologically rigorous studies have determined the long-term effects of COVID-19 in primary care patients. A Norwegian prospective cohort study of 8,786 adults tested for SARS-CoV-2 (794 positive and 7229 negative) found that 36% of SARS-CoV-2 positive participants reported worse health than a year ago at 3–8 months follow-up compared to 18% of negative participants, with significant differences in smell and taste changes and ‘other symptoms’ [10]. A Swedish study including 1395 healthcare professionals found persisting symptoms more frequently in serology-positive participants at 8 months follow-up [18]. An Italian study on 150 healthcare workers found no significant difference in neurological deficits and cognitive impairment at 4 months between those with and without confirmed SARS-CoV-2 infection [19]. These studies, however, had shorter follow-up periods and participants were aware of their SARS-CoV-2 status. Besides, controls did not necessarily had experienced a respiratory tract infection.

In a French population-based cohort, persisting symptoms at 10-12 months after the first COVID-19 wave were more frequent in self-reported COVID-19 cases, but differences diminished, except for anosmia, when comparing serology positive and negative participants [9]. This suggests that the belief in having been infected with SARS-CoV-2 may influence the occurrence of persisting symptoms following COVID-19.

In a more recent publication of the PANORAMIC trial, 11% (946/8634) of UK participants who were randomly allocated to usual care reported any persisting symptom 3–6 months following randomisation [20]. This trial was however conducted in the Omicron era (December 2021–April 2022) and included a largely vaccinated population at higher risk of more severe outcome which hampers direct comparison with our study population.

A Scottish cohort study found 65% of adults reported at least one symptom six months post-infection compared to 51% of matched never-infected adults, with differences in symptom prevalence decreasing from 14% to 7% after adjusting for confounders [21]. These findings are consistent with our results, which show no significant difference in the resolution of symptoms during 12 months follow-up between individuals with and without confirmed SARS-CoV-2 infection. The comparable HRQoL profiles between SARS-CoV-2 serology-positive and negative participants over 12 months following moderately severe LRTI suggest that long-term sequelae might not be specific to SARS-CoV-2.

To our knowledge, this is the first study in which participants, healthcare professionals as well as study personnel were blinded to whether study participants had a SARS-CoV-2 infection or not. In addition, at the time of the start of the study, both clinicians and patients were still unaware of the nature of the disease COVID-19 and its potential long term sequelae. This is important as blinding prevents biases, such as information and recall bias. Another strength is the prospective design and inclusion of a SARS-CoV-2 serology-negative control group with a standardised and detailed collection of important prognostic factors (confounders).

A potential limitation is the misclassification of SARS-CoV-2 status due to waning antibodies. However, IgG remains above the seropositivity threshold in about 92% of patients after 7 months, likely even more so in patients with severe symptoms [22]. Additionally, higher antibody titres are associated with more persistent symptoms, reducing the likelihood of misclassification of COVID-19 patients with persistent symptoms [7]. Therefore, we believe that this did not substantially influence our findings.

Some patients might have acquired an asymptomatic SARS-CoV-2 infection after the index consultation, potentially diluting the contrast between groups. Patients were asked in each questionnaire about any SARS-CoV-2 infections post-index consultation, so most infections were registered, though asymptomatic ones could have been missed. An open population study during the same period showed a point-prevalence of asymptomatic COVID-19 patients in outpatients of only about 3% [23].

Participants provided data about the index consultation, which occurred on average seven months prior. This may have led to inaccuracies in recalling HRQoL and symptoms at that time. However, HRQoL modelling was mostly based on prospective data, with only the SF-36 scores from 2 weeks prior to the index consultation determined retrospectively. Due to blinding, it is unlikely that recall inaccuracies differed significantly between SARS-CoV-2 serology positive and negative participants. Finally, our study population was not vaccinated against COVID-19 and probably not previously infected by SARS-CoV-2 at the time of the index consultation. It remains unclear whether the presence of SARS-CoV-2 antibodies, through vaccination or acquired infection would lead to fewer persisting symptoms. Additionally, our conclusions apply specifically to patients with moderately severe lower respiratory tract infections treated in primary care.

Our results indicate that persisting symptoms occur in a substantial proportion of patients following an LRTI. Observed long-term sequelae after moderately severe SARS-CoV-2 LRTI in primary care may not be specific to SARS-CoV-2. Informing patients about possible long term sequelae and offering support and management to those patients seems indicated. Further studies investigating the persistence of symptoms after LRTIs caused by different pathogens are therefore warranted.

## Conclusion

No clear differences in HRQoL or symptomatology were observed between primary care treated patients with and without confirmed SARS-CoV-2 infection following moderately severe LRTI over the first year. A substantial proportion of patients reported symptoms beyond 4 weeks, indicating that persisting symptoms might not be specific to SARS-CoV-2 and can occur in LRTIs caused by other pathogens. Further research should focus on persisting symptoms after LRTIs from various pathogens.

## Supplementary Material

Supplemental Material

## Data Availability

Individual participant data collected during the study will be available, after deidentification of all participants. Data will be available to researchers who provide a methodologically sound proposal to achieve the aims in the approved proposal. Proposals should be directed to the corresponding author to gain access to the data. Data requestors will need to sign a data sharing agreement.

## Transparency

The lead author (the manuscript’s guarantor) affirms that the manuscript is an honest, accurate, and transparent account of the study being reported; that no important aspects of the study have been omitted; and that any discrepancies from the study as planned (and, if relevant, registered) have been explained.
