# Supplementary material for: Long-term prognosis of adults with moderately severe SARS-CoV-2 lower respiratory tract infection managed in primary care: Prospective cohort study
Source: Eur J Gen Pract. 2025 Jun 2;31(1):2501306. doi: 10.1080/13814788.2025.2501306 (PMC12131542; doi:10.1080/13814788.2025.2501306)
Supplement: Supplemental Material [file IGEN_A_2501306_SM8918.zip › IGEN_A_2501306_suppl_data/ejgp-2024-0225-File002.docx]

*Figure 1 – Timeline index consultation, baseline and follow-up measurements*


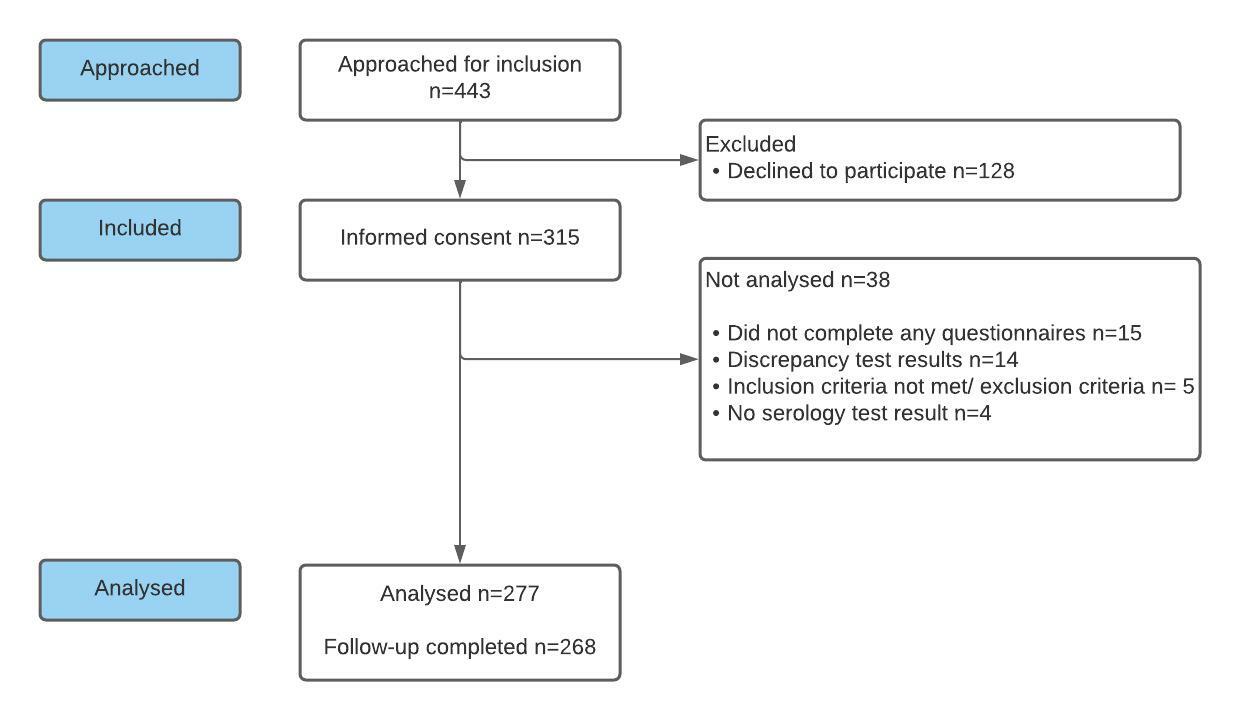


*Figure 2 – Patient selection flowchart*


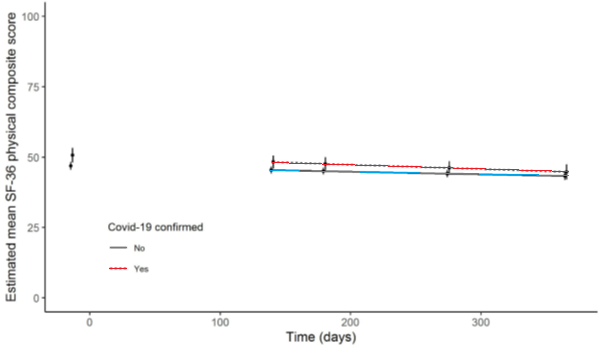


Estimated differences: negative versus positive SARS-CoV-2 serology test result 0-90 days: 0.55 (95%CI -0.16 - 1.25); 0-180 days: 1.10 (95% CI -0.31 - 2.50); 0-270 days: 1.64 (95%CI -0.47 - 3.75) and 0-360 days: 2.22 (95%CI -0.63 - 5.07).

*Figure 3 Estimated mean Physical Component Summary (PCS) score (SF-36) over time, based on mixed model.*

*
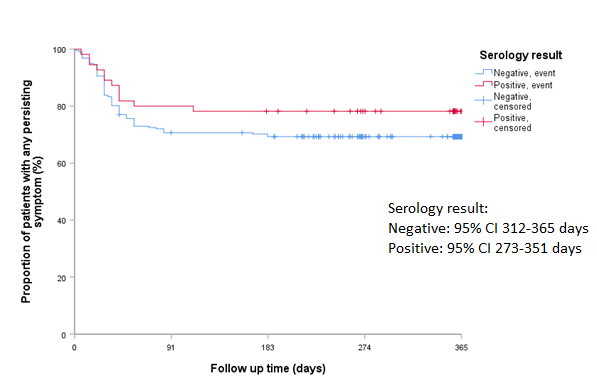
*

*Figure 4 Kaplan Meier curve of the proportion of patients with any persisting symptom over the 12 month follow up*
