# Supplementary material for: Long-term prognosis of adults with moderately severe SARS-CoV-2 lower respiratory tract infection managed in primary care: Prospective cohort study
Source: Eur J Gen Pract. 2025 Jun 2;31(1):2501306. doi: 10.1080/13814788.2025.2501306 (PMC12131542; doi:10.1080/13814788.2025.2501306)
Supplement: Supplemental Material [file IGEN_A_2501306_SM8918.zip › IGEN_A_2501306_suppl_data/ejgp-2024-0225-File003.docx]

Appendix S1

Detailed Methods

*Inclusion of patients*

Potentially eligible participants were informed about the study by their GP, and those interested received verbal and written information from the study team.

Study Procedures, Baseline and Follow-up Measurements

*The SF-36* HRQoL questionnaire consists of a single item of health transition (HT) and a further 35 items which can be divided into eight subscales: (1) physical function (PF), (2) limitations due to physical health problems (role physical, RP), (3) bodily pain (BP), (4) general health (GH), (5) vitality (VT), (6) social functioning (SF), (7) limitations due to emotional health problems (RE), and (8) mental health (MH). Scores range from 0 to 100, with higher scores indicating better functional status. The eight subdomain scores were aggregated into two summary measures: physical component summary (PCS) and mental component summary (MCS) scores.

Statistical Analyses

Baseline characteristics were compared between those with and without established SARS-CoV-2 infection (based on serology testing) using appropriate statistical tests. For the primary analysis, a linear mixed effects model was used with SF-36 PSC scores as the dependent variable and SARS-CoV-2 serology test result, time, age, gender, body mass index (BMI), diabetes, chronic pulmonary diseases, and the interaction term between time and SARS-CoV-2 serology test result as fixed effects. A random intercept and slope for time accounted for repeated measurements. The difference in change of SF-36 PSC score from 2 weeks prior to 12 months after the index consultation between serology-positive and negative groups was estimated with 95% confidence intervals (CIs). Differences in changes in SF-36 MCS scores and the eight individual SF-36 subscales between groups were estimated similarly.

For time to symptom resolution, Kaplan-Meier survival analyses with log-rank tests were performed. Participants with persisting symptoms were censored at the end of follow-up. Unadjusted hazard ratios (HRs) with 95% CIs were calculated using Cox proportional hazard modeling, adjusting for age, gender, and comorbidities (chronic pulmonary disease, cardiovascular disease, and diabetes). The proportional hazards assumption was tested using the supremum test, adding time-dependent coefficients when violated. Effect modification by chronic pulmonary disease, cardiovascular disease, and diabetes was assessed by adding interaction terms with SARS-CoV-2 serology test result.

Several assumptions were made: the study SARS-CoV-2 serology test results were used to attribute moderately severe LRTI at index consultation to SARS-CoV-2 infection, excluding participants with discrepancies between study and routine care RT-PCR or serology tests. Participants testing RT-PCR positive between one month after the index consultation and 14 days before the study serology were considered SARS-CoV-2 negative until the positive RT-PCR result. Participants with a negative serology test who experienced a SARS-CoV-2 infection during follow-up were censored from the moment of the positive test.

Data were analyzed using IBM SPSS Statistics version 26.0.0.1 and SAS version 9.4 for the linear mixed effects model and the proportional hazards checks. Statistical significance was assumed at p <0.05.
