# Supplementary material for: Long-term prognosis of adults with moderately severe SARS-CoV-2 lower respiratory tract infection managed in primary care: Prospective cohort study
Source: Eur J Gen Pract. 2025 Jun 2;31(1):2501306. doi: 10.1080/13814788.2025.2501306 (PMC12131542; doi:10.1080/13814788.2025.2501306)

Appendix S2

Estimated differences: negative versus positive SARS-CoV-2 serology test result 0-90 days: -0.37 (95%CI -1.06 – 0.33); 0-180 days: -0.73 (95% CI -2.12 – 0.66); 0-270 days: -1.10 (95%CI -3.19 – 0.99) and 0-360 days: -1.48 (95%CI -4.31 – 1.34)

*Figure appendix S2 Estimated mean MCS score (SF-36) over time, based on mixed model.*

Appendix S3

*Appendix S3 Kaplan Meier survival curves of proportion of patients with persisting (A) shortness of breath, (B) cough, (C) fatigue, (D) brain fog, (E) anosmia/ageusia, (F) headache and (G) chest pain over a 12 month follow up*

A B


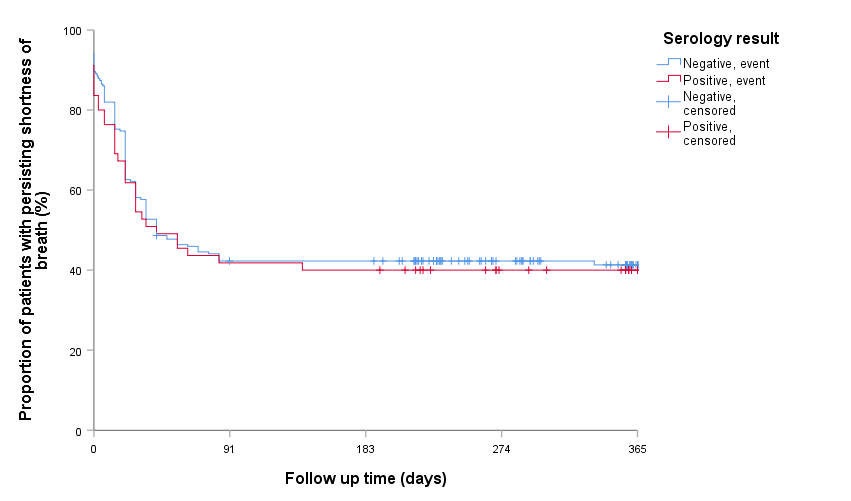

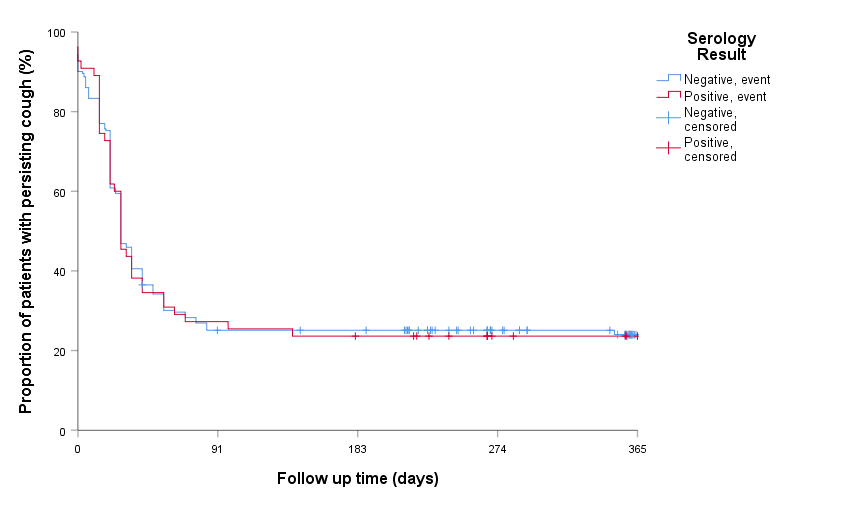


C D


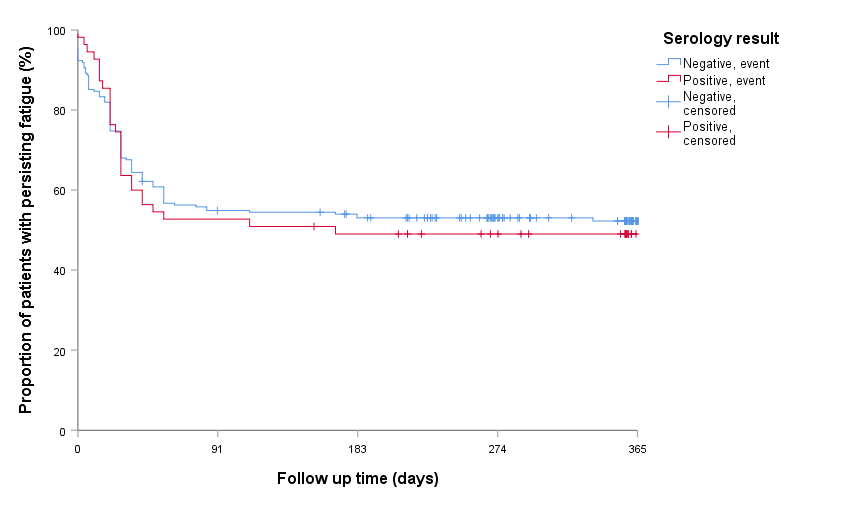

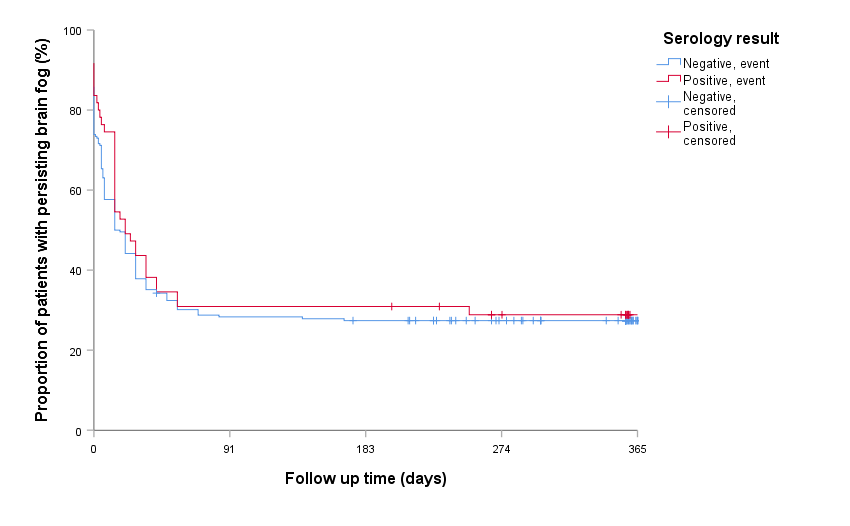


E F


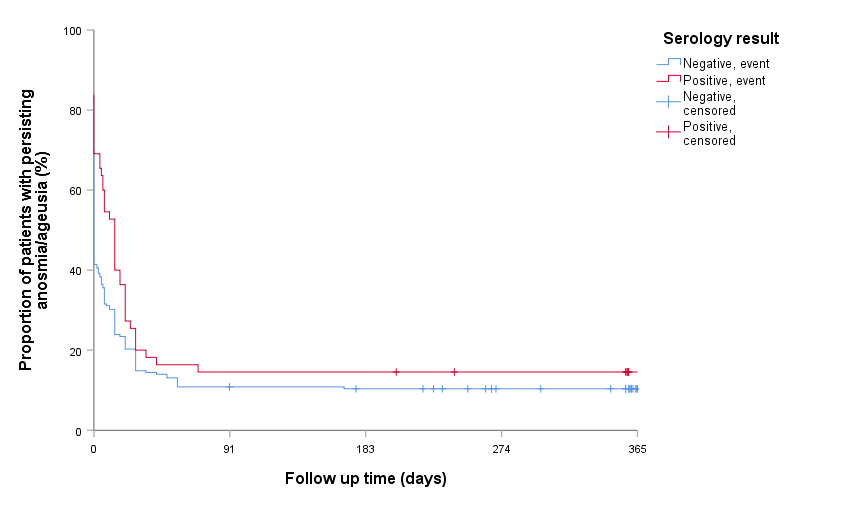

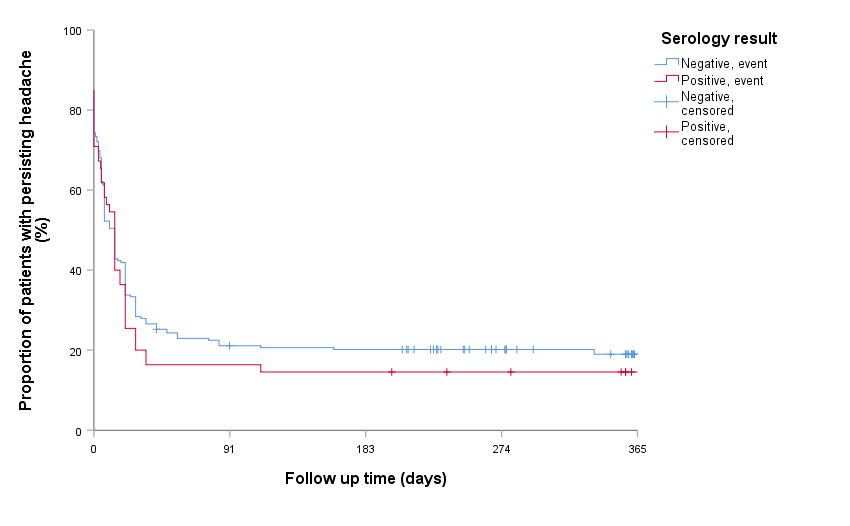


G


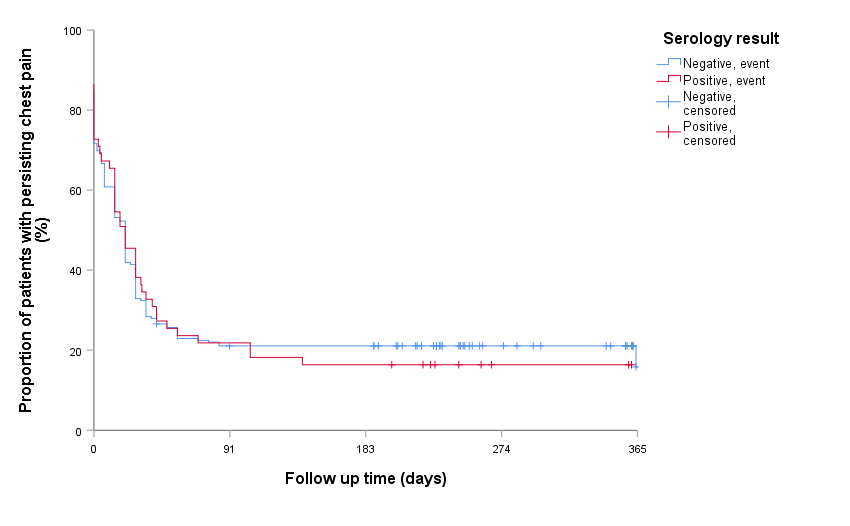

Supplement: Supplemental Material [file IGEN_A_2501306_SM8918.zip › IGEN_A_2501306_suppl_data/ejgp-2024-0225-File004.docx]
