# Supplementary material for: Long-term prognosis of adults with moderately severe SARS-CoV-2 lower respiratory tract infection managed in primary care: Prospective cohort study
Source: Eur J Gen Pract. 2025 Jun 2;31(1):2501306. doi: 10.1080/13814788.2025.2501306 (PMC12131542; doi:10.1080/13814788.2025.2501306)
Supplement: Supplemental Material [file IGEN_A_2501306_SM8918.zip › IGEN_A_2501306_suppl_data/tejp-2024-0118-File010.docx]

**TABLE S1 *Ulva* species type specimen sequences used to populate the kraken2 database for determining species identification. Note: some specimens included in the database were not sequenced from type specimens due to unavailability and/or uncertainty DNA of those *Ulva* species.**

| **Species name** | ***rbc*L** | **ITS I** | ***tuf*A** | **Reference** |
| --- | --- | --- | --- | --- |
| *Ulva adhaerens* | AB894327 | AB894334 |  | Matsumoto et al. 2015 |
| *Ulva australis* | LC331300 | LC331301 | MT160674 | Fort et al. 2022; Hanyuda and Kawai, 2018; Hughey et al. 2022 |
| *Ulva brisbanensis* | EU933945 | EU933972 |  | Kraft et al. 2010 |
| *Ulva californica* | OR723827 | OR722833 | OR723828 | Maggs et al. 2024 |
| *Ulva chaugulii* | KP710829 | KT429218 |  | Kazi et al. 2016 |
| *Ulva clathrata* |  | OR734717 |  | Maggs et al. 2024 |
| *Ulva compressa* | OR427286 | OR734719 | OR427287 | Maggs et al. 2024 |
| *Ulva conglobata* | MT815850 |  |  | Hughey et al. 2021 |
| *Ulva expansa* | MH731009 | MH730160 | MH731007 | Hughey et al. 2018 |
| *Ulva fasciata* synonymous with U. lactuca* | MK456397 |  |  | Hughey et al. 2019 |
| *Ulva fenestrata* | MK456393 |  | MK456404 | Hughey et al. 2019 |
| *Ulva iliohaha* | KT932995 | KT881223 | KT932976 | Spalding et al. 2019 |
| *Ulva intestinalis* | AY255860 | OR734710 | MZ561630 | Hayden et al. 2003; Wang et al. 2021; Maggs et al. 2024 |
| *Ulva kraftiorum* | EU933963 | EU933991 |  | Kraft et al. 2010 |
| *Ulva lacinulata* | MW543061 | MW544060 | MW543061 | Hughey et al. 2021 |
| *Ulva lactuca* | MK456395 |  | KM212026 | Bertuccio et al. 2014; Hughey et al. 2019 |
| *Ulva limnetica* | AB425968 |  |  | Ichihara et al. 2009 |
| *Ulva linza* | OR766869 | OR734711 | HQ610368 | Maggs et al, 2024 |
| *Ulva meridionalis* | AB598812 |  |  | Horimoto et al. 2011 |
| *Ulva ohiohilulu* |  | KT881224 | KT932977 | Spalding et al. 2016 |
| *Ulva ohnoi* | AP018696 | AB116031 | OR723884 | Hiraoka et al. 2004; Suzuki et al. 2018; Hughey et al. 2024 |
| *Ulva partita* | LC021415 |  |  | Ichihara et al. 2015 |
| *Ulva pilifera* | HM447571 |  |  | Mareš et al. 2011 |
| *Ulva prolifera* | AY255864 | OR734712 | HQ610402 | Saunders and Kucera, 2010; Hayden et al. 2003; Maggs et al. 2024 |
| *Ulva pseudocurvata* | OR635440 | OR536260 | OR539204 | Maggs et al. 2024 |
| *Ulva pseudohnoi* | MT624844 |  | MT625015 | Lee et al. 2019 |
| *Ulva rigida* | MW543060 | MW544059 | MW543060 | Hughey et al. 2021 |
| *Ulva shanxiensis* |  |  | KJ617036 | Chen et al. 2015 |
| *Ulva stenophylloides* | EU933955 | EU933983 |  | Kraft et al. 2010 |
| *Ulva torta* | ON512837 |  | OL421272 | Bartolo et al. 2022 |
| *Ulva uncialis* | OL778910 | OM146563 | OL778904 | Bachoo et al. 2023 |
| *Ulva uniseriata* |  | KX668899 |  | Bast and Rani, 2019 |
| *Ulvaria obscura* | OR766878 | OR734720 | OR766888 | Maggs et al. 2024 |
| *Ulvaria splendens* | OR491123 |  | OR539203 | Maggs et al. 2024 |


**References**

Bachoo, T., Bolton, J.J., Macey, B.M., Kandjengo, L. & Reddy, M.M. (2023). Resolving the identity of commercially cultivated *Ulva* (Ulvaceae, Chlorophyta) in integrated seaweed‐abalone aquaculture farms in South Africa. *Journal of Phycology*, **59:** 1272-1283.

Bartolo, A. G., Zammit, G., & Küpper, F. C. (2022). *Ulva* L. biodiversity in the central Mediterranean Sea: cryptic species and new records. *Cryptogamie, Algologie*, **43**: 215-225.

Bast, F., & Rani, P. (2019). First report of uniseriate free-living *Ulva* species with description of new species *Ulva uniseriata* sp. nov (Chlorophyta, Ulvales). *Indian Journal of Geo Marine Sciences*, **48**: 1687-1691.

Bertuccio, C., Genovese, G., Manghisi, A., Cruaud, C., Couloux, A., Le Gall, L., & Morabito, M. (2014). Changes in the benthic algal flora of Lake Ganzirri, North-Eastern Sicily (Italy). *Natura rerum*, **3**: 79-91.

Bringloe, T.T., Dunton, K.H. and Saunders, G.W.(2017). Updates to the marine algal flora of the Boulder Patch in the Beaufort Sea off Northern Alaska as revealed by DNA Barcoding. *Arctic*, **70***:*343-348.

Fort, A., Linderhof, C., Coca-Tagarro, I., Inaba, M., McHale, M., Cascella, K., Potin, P., Guiry, M.D., & Sulpice, R. (2021). A sequencing-free assay for foliose *Ulva* species identification, hybrid detection and bulk biomass characterisation. *Algal Research,* **55**: 102280.

Chen, L., Feng, J., & Xie, S. L. (2015). *Ulva* *shanxiensis* (Ulvaceae), a new species from Shanxi, China. *Novon: A Journal for Botanical Nomenclature*, **23**: 397-405.

Gadberry, B.A., Colt, J., Maynard, D., Boratyn, D.C., Webb, K., Johnson, R.B., Saunders, G.W. and Boyer, R.H. (2018). Intensive land-based production of red and green macroalgae for human consumption in the Pacific Northwest: an evaluation of seasonal growth, yield, nutritional composition, and contaminant levels. *Algae*, **33**:109-125.

Hanyuda, T. & Kawai, H. (2018). Genetic examination of the type specimen of *Ulva australis* suggests that it was introduced to Australia. *Phycological Research*, **66**: 238–241.

Hayden, H. S., Blomster, J., Maggs, C. A., Silva, P. C., Stanhope, M. J., & Waaland, J. R. (2003). Linnaeus was right all along: *Ulva* and *Enteromorpha* are not distinct genera. *European Journal of Phycology*, **38**: 277-294.

Hiraoka, M., Shimada, S., Uenosono, M., & Masuda, M. (2004). A new green‐tide‐forming alga, *Ulva ohnoi* Hiraoka et Shimada sp. nov. (Ulvales, Ulvophyceae) from Japan. *Phycological research*, ***52***:17-29.

Horimoto, R., Masakiyo, Y., & Ichihara, K. (2011). *Enteromorpha*-like *Ulva* (Ulvophyceae, Chlorophyta) growing in the Todoroki River, Ishigaki Island, Japan, with special reference to *Ulva meridionalis* Horimoto et Shimada, sp. nov. *Bulletin of the National Museum of Nature and Science: Botany,* **37***:* 155-167.

Hughey, J.R., Miller, K.A. and Gabrielson, P.W.(2018). Mitogenome analysis of a green tide forming *Ulva* from California, USA confirms its identity as *Ulva expansa* (Ulvaceae, Chlorophyta). *Mitochondrial DNA Part B*, **3**:1302-1303.

Hughey, J. R., Maggs, C. A., Mineur, F., Jarvis, C., Miller, K. A., Shabaka, S. H., & Gabrielson, P. W. (2019). Genetic analysis of the Linnaean *Ulva lactuca* (Ulvales, Chlorophyta) holotype and related type specimens reveals name misapplications, unexpected origins, and new synonymies. *Journal of Phycology*, **55**:503-508.

Hughey, J. R., Gabrielson, P. W., Maggs, C. A. & Mineur, F. (2021). Genomic analysis of the lectotype specimens of European *Ulva rigida* and *Ulva lacinulata* (Ulvaceae, Chlorophyta) reveals the ongoing misapplication of names. *European Journal of Phycology*, **57**: 143-153.

Ichihara, K., Arai, S., Uchimura, M., Fay, E. J., Ebata, H., Hiraoka, M., & Shimada, S. (2009). New species of freshwater *Ulva*, *Ulva limnetica* (Ulvales, Ulvophyceae) from the Ryukyu Islands, Japan. *Phycological Research,* **57**: 94-103.

Ichihara, K., Suzuki, R., Yamazaki, T., Ota, S., Mogi, Y., Kagami, Y., ... & Kawano, S. (2015). *Ulva partita* sp. nov., a novel *Enteromorpha*-like *Ulva* species from Japanese coastal areas. *Cytologia*, **80**: 261-270.

Kang, J.H., Jang, J.E., Kim, J.H., Byeon, S.Y., Kim, S., Choi, S.K., Kang, Y.H., Park, S.R. and Lee, H.J.(2019). Species composition, diversity, and distribution of the genus *Ulva* along the coast of Jeju Island, Korea based on molecular phylogenetic analysis. *PloS one*, **14**: 0219958.

Kazi, M. A., Kavale, M. G., & Singh, V. V. (2016). Morphological and molecular characterization of *Ulva chaugulii* sp. nov., *U. lactuca* and *U. ohnoi* (Ulvophyceae, Chlorophyta) from India. *Phycologia*, **55**: 45-54.

Kirkendale, L., Saunders, G. W., & Winberg, P. (2013). A molecular survey of *Ulva* (Chlorophyta) in temperate Australia reveals enhanced levels of cosmopolitanism. *Journal of Phycology*, **49***:*69-81.

Kraft, L. G., Kraft, G. T., & Waller, R. F. (2010). Investigations into Southern Australian *Ulva* (Ulvophyceae, Chlorophyta) taxonomy and molecular phylogeny indicate both cosmopolitanism and endemic cryptic species 1. *Journal of Phycology*, **46**: 1257-1277.

Lee, H. W., Kang, J. C., Kim, M. S., Lee, H. W., Kang, J. C., & Kim, M. S. (2019). Taxonomy of *Ulva* causing blooms from Jeju Island, Korea with new species, *U. pseudo-ohnoi* sp. nov. (Ulvales, Chlorophyta). *Algae*, **34**: 253-266.

Maggs, C., Bunker, A., Bunker, F., Harries, D., Kelly, J., Mineur, F., Blomster, J., Díaz-Tapia, P., Gabrielson, P., Hughey, J. & Brodie, J. (2024). Updating the Ulvaceae in the green seaweeds of Britain and Ireland. *Botanica Marina*, **67**: 181-203.

Mareš, J., Leskinen, E., Sitkowska, M., Skácelová, O., & Blomster, J. (2011). True identity of the European freshwater *Ulva* (Chlorophyta, Ulvophyceae) revealed by a combined molecular and morphological approach. *Journal of Phycology*, **47**: 1177-1192.

Matsumoto, K., & Shimada, S. (2015). Systematics of green algae resembling *Ulva conglobata*, with a description of *Ulva adhaerens* sp. nov. (Ulvales, Ulvophyceae). *European Journal of Phycology*, **50**: 100-111.

Saunders, G.W. and Kucera, H. (2010). An evaluation of *rbc*L, *tuf*A, UPA, LSU and ITS as DNA barcode markers for the marine green macroalgae. *Cryptogamie, Algologie*, **31**: 487-528.

Spalding, H.L., Conklin, K.Y., Smith, C.M., O'Kelly, C.J. and Sherwood, A.R. (2016). New Ulvaceae (Ulvophyceae, Chlorophyta) from mesophotic ecosystems across the Hawaiian archipelago. *Journal of Phycology*, **52:** 40-53.

Spalding, H. L., Amado-Filho, G. M., Bahia, R. G., Ballantine, D. L., Fredericq, S., Leichter, J. J., ... & Tsuda, R. T. (2019). Macroalgae. In: Loya, Y., Puglise, K., Bridge, T. (eds) Mesophotic Coral Ecosystems. *Coral Reefs of the World*, **12**: 507-536. Springer.

Steinhagen, S., Karez, R. and Weinberger, F.(2019). Cryptic, alien and lost species: molecular diversity of *Ulva sensu* lato along the German coasts of the North and Baltic Seas. *European Journal of Phycology*, **54:** 466-483.

Steinhagen, S., Barco, A., Wichard, T. and Weinberger, F.(2019). Conspecificity of the model organism *Ulva mutabilis* and *Ulva compressa* (Ulvophyceae, Chlorophyta). *Journal of Phycology*, **55:** 25-36.

Suzuki, S., Yamaguchi, H., Hiraoka, M., & Kawachi, M. (2018). Mitochondrial and chloroplast genome sequences of *Ulva ohnoi*, a green-tide-forming macroalga in the Southern coastal regions of Japan. *Mitochondrial DNA Part B*, **3**: 765-767.

Wang, H., Liu, F., Wang, J., & Chen, N. (2021). Phylogenomic analysis of the chloroplast genome of the green-tide forming macroalga *Ulva intestinalis* Linnaeus (Ulvophyceae, Chlorophyta). *Mitochondrial DNA Part B*, **6**: 3052-3054.

Xie, W.F., Wu, C.H., Zhao, J., Lin, X.Y. and Jiang, P.(2020). New records of *Ulva* spp. (Ulvophyceae, Chlorophyta) in China, with special reference to an unusual morphology of *U. meridionalis* forming green tides. *European Journal of Phycology*, **55**: 412-425.

**TABLE S2** Metadata for NGS and CAPS analysed specimens SRA bioproject PRJNA1014254 collected and described in this study.

| **Identification** | **Date Collected** | **Locality** | **Sample Number** | **LAT** | **LONG** | **Mat Density** | **Site Characteristics** | **Analysis (%)** |
| --- | --- | --- | --- | --- | --- | --- | --- | --- |
| *Chaetomorpha ligustica* | 25/03/2021 | Langstone Harbour, UK | L250321SA2 | 50°47'36.51"N | 1° 1'50.57"W | High | Silt to gravel mudflat sediment type, adjacent seagrass, salt marsh habitats | NGS (89.8) |
| *Chaetomorpha linum* | 25/03/2021 | Langtsone Harbour, UK | L250321SA2 | 50°47'36.51"N | 1° 1'50.57"W | High | Silt to gravel mudflat sediment type, adjacent seagrass, salt marsh habitats | NGS (3.8) |
| *Pyllaiella littoralis* | 25/03/2021 | Langstone Harbour, UK | L250321SA2 | 50°47'36.51"N | 1° 1'50.57"W | High | Silt to gravel mudflat sediment type, adjacent seagrass, salt marsh habitats | NGS (6.38) |
| *Chaetomorpha ligustica* | 25/03/2021 | Langstone Harbour, UK | L250321SA3 | 50°47'36.33"N | 1° 1'50.34"W | Med | Silt to gravel mudflat sediment type, adjacent seagrass, salt marsh habitats | NGS (76.2) |
| *Chaetomorpha linum* | 25/03/2021 | Langstone Harbour, UK | L250321SA3 | 50°47'36.33"N | 1° 1'50.34"W | Med | Silt to gravel mudflat sediment type, adjacent seagrass, salt marsh habitats | NGS (10.48) |
| *Pyllaiella littoralis* | 25/03/2021 | Langstone Harbour, UK | L250321SA3 | 50°47'36.33"N | 1° 1'50.34"W | Med | Silt to gravel mudflat sediment type, adjacent seagrass, salt marsh habitats | NGS (13.3) |
| *Chaetomorpha ligustica* | 25/03/2021 | Langstone Harbour, UK | L250321SA8 | 50°47'36.78"N | 1° 1'50.56"W | Low | Silt to gravel mudflat sediment type, adjacent seagrass, salt marsh habitats | NGS (83.4) |
| *Chaetomorpha linum* | 25/03/2021 | Langstone Harbour, UK | L250321SA8 | 50°47'36.78"N | 1° 1'50.56"W | Low | Silt to gravel mudflat sediment type, adjacent seagrass, salt marsh habitats | NGS (3) |
| *Pyllaiella littoralis* | 25/03/2021 | Langstone Harbour, UK | L250321SA8 | 50°47'36.78"N | 1° 1'50.56"W | Low | Silt to gravel mudflat sediment type, adjacent seagrass, salt marsh habitats | NGS (13.5) |
| *Ulva lacinulata* | 16/06/2021 | Langstone Harbour, UK | L160621SA_high | 50°47'37.46"N | 1° 1'50.98"W | High | Silt to gravel mudflat sediment type, adjacent seagrass, salt marsh habitats | NGS (53.1) |
| *Ulva clathrata* | 16/06/2021 | Langstone Harbour, UK | L160621SA_high | 50°47'37.46"N | 1° 1'50.98"W | High | Silt to gravel mudflat sediment type, adjacent seagrass, salt marsh habitats | NGS (2.5) |
| *Cladophora aldiba* | 16/06/2021 | Langstone Harbour, UK | L160621SA_high | 50°47'37.46"N | 1° 1'50.98"W | High | Silt to gravel mudflat sediment type, adjacent seagrass, salt marsh habitats | NGS (44.3) |
| *Ulva lacinulata* | 16/06/2021 | Langstone Harbour, UK | L160621SA_med | 50°47'37.92"N | 1° 1'51.56"W | Med | Silt to gravel mudflat sediment type, adjacent seagrass, salt marsh habitats | NGS (46.4) |
| *Ulva clathrata* | 16/06/2021 | Langstone Harbour, UK | L160621SA_med | 50°47'37.92"N | 1° 1'51.56"W | Med | Silt to gravel mudflat sediment type, adjacent seagrass, salt marsh habitats | NGS (5.9) |
| *Cladophora aldiba* | 16/06/2021 | Langstone Harbour, UK | L160621SA_med | 50°47'37.92"N | 1° 1'51.56"W | Med | Silt to gravel mudflat sediment type, adjacent seagrass, salt marsh habitats | NGS (30.5) |
| *Pylaiella littoralis* | 16/06/2021 | Langstone Harbour, UK | L160621SA_med | 50°47'37.92"N | 1° 1'51.56"W | Med | Silt to gravel mudflat sediment type, adjacent seagrass, salt marsh habitats | NGS (11) |
| *Ulva sp. LPP* | 16/06/2021 | Langstone Harbour, UK | L160621SA_med | 50°47'37.92"N | 1° 1'51.56"W | Med | Silt to gravel mudflat sediment type, adjacent seagrass, salt marsh habitats | NGS (6) |
| *Ulva lacinulata* | 16/06/2021 | Langstone Harbour, UK | L160621SA_low | 50°47'39.46"N | 1° 1'51.84"W | Low | Silt to gravel mudflat sediment type, adjacent seagrass, salt marsh habitats | NGS (55.6) |
| *Ulva clathrata* | 16/06/2021 | Langstone Harbour, UK | L160621SA_low | 50°47'39.46"N | 1° 1'51.84"W | Low | Silt to gravel mudflat sediment type, adjacent seagrass, salt marsh habitats | NGS (9.2) |
| *Ulva sp. LPP* | 16/06/2021 | Langstone Harbour, UK | L160621SA_low | 50°47'39.46"N | 1° 1'51.84"W | Low | Silt to gravel mudflat sediment type, adjacent seagrass, salt marsh habitats | NGS (2.5) |
| *Cladophora albida* | 16/06/2021 | Langstone Harbour, UK | L160621SA_low | 50°47'39.46"N | 1° 1'51.84"W | Low | Silt to gravel mudflat sediment type, adjacent seagrass, salt marsh habitats | NGS (19.7) |
| *Cladophora laetevirens* | 16/06/2021 | Langstone Harbour, UK | L160621SA_low | 50°47'39.46"N | 1° 1'51.84"W | Low | Silt to gravel mudflat sediment type, adjacent seagrass, salt marsh habitats | NGS (4.8) |
| *Chaetomorpha ligustica* | 16/06/2021 | Langstone Harbour, UK | L160621SA_low | 50°47'39.46"N | 1° 1'51.84"W | Low | Silt to gravel mudflat sediment type, adjacent seagrass, salt marsh habitats | NGS (3.8) |
| *Chaetomorpha linum* | 16/06/2021 | Langstone Harbour, UK | L160621SA_low | 50°47'39.46"N | 1° 1'51.84"W | Low | Silt to gravel mudflat sediment type, adjacent seagrass, salt marsh habitats | NGS (2) |
| *Pylaiella littoralis* | 16/06/2021 | Langstone Harbour, UK | L160621SA_low | 50°47'39.46"N | 1° 1'51.84"W | Low | Silt to gravel mudflat sediment type, adjacent seagrass, salt marsh habitats | NGS (1.5) |
| *Cladophora albida* | 13/09/2021 | Langstone Harbour, UK | L130921SA LD | 50°47'37.86"N | 1° 1'52.23"W | Low | Silt to gravel mudflat sediment type, adjacent seagrass, salt marsh habitats | NGS (31.8) |
| *Cladophora laetevirens* | 13/09/2021 | Langstone Harbour, UK | L130921SA LD | 50°47'37.86"N | 1° 1'52.23"W | Low | Silt to gravel mudflat sediment type, adjacent seagrass, salt marsh habitats | NGS (36.1) |
| *Ulva clathrata* | 13/09/2021 | Langstone Harbour, UK | L130921SA LD | 50°47'37.86"N | 1° 1'52.23"W | Low | Silt to gravel mudflat sediment type, adjacent seagrass, salt marsh habitats | NGS (18.6) |
| *Chaetomorpha linum* | 13/09/2021 | Langstone Harbour, UK | L130921SA LD | 50°47'37.86"N | 1° 1'52.23"W | Low | Silt to gravel mudflat sediment type, adjacent seagrass, salt marsh habitats | NGS (10.1) |
| *Chaetomorpha ligustica* | 13/09/2021 | Langstone Harbour, UK | L130921SA LD | 50°47'37.86"N | 1° 1'52.23"W | Low | Silt to gravel mudflat sediment type, adjacent seagrass, salt marsh habitats | NGS (3.3) |
| *Cladophora albida* | 13/09/2021 | Langstone Harbour, UK | L130921SA MD | Not taken |  | Med | Silt to gravel mudflat sediment type, adjacent seagrass, salt marsh habitats | NGS (18.5) |
| *Cladophora laetevirens* | 13/09/2021 | Langstone Harbour, UK | L130921SA MD | Not taken |  | Med | Silt to gravel mudflat sediment type, adjacent seagrass, salt marsh habitats | NGS (25) |
| *Ulva clathrata* | 13/09/2021 | Langstone Harbour, UK | L130921SA MD | Not taken |  | Med | Silt to gravel mudflat sediment type, adjacent seagrass, salt marsh habitats | NGS (32.9) |
| *Chaetomorpha linum* | 13/09/2021 | Langstone Harbour, UK | L130921SA MD | Not taken |  | Med | Silt to gravel mudflat sediment type, adjacent seagrass, salt marsh habitats | NGS (19.7) |
| *Chaetomorpha ligustica* | 13/09/2021 | Langstone Harbour, UK | L130921SA MD | Not taken |  | Med | Silt to gravel mudflat sediment type, adjacent seagrass, salt marsh habitats | NGS (3.4) |
| *Cladophora albida* | 13/09/2021 | Langstone Harbour, UK | L130921SA HD | Not taken |  | High | Silt to gravel mudflat sediment type, adjacent seagrass, salt marsh habitats | NGS (36.2) |
| *Cladophora laetevirens* | 13/09/2021 | Langstone Harbour, UK | L130921SA HD | Not taken |  | High | Silt to gravel mudflat sediment type, adjacent seagrass, salt marsh habitats | NGS (43.1) |
| *Chaetomorpha linum* | 13/09/2021 | Langstone Harbour, UK | L130921SA HD | Not taken |  | High | Silt to gravel mudflat sediment type, adjacent seagrass, salt marsh habitats | NGS (8.7) |
| *Chaetomorpha ligustica* | 13/09/2021 | Langstone Harbour, UK | L130921SA HD | Not taken |  | High | Silt to gravel mudflat sediment type, adjacent seagrass, salt marsh habitats | NGS (5) |
| *Ulva clathrata* | 13/09/2021 | Langstone Harbour, UK | L130921SA HD | Not taken |  | High | Silt to gravel mudflat sediment type, adjacent seagrass, salt marsh habitats | NGS (6.8) |
| *Chaetomorpha ligustica* | 16/12/2021 | Langstone Harbour, UK | L161221SA LD | 50°47'37.71"N | 1° 1'50.91"W | Low | Silt to gravel mudflat sediment type, adjacent seagrass, salt marsh habitats | NGS (2.7) |
| *Cladophora laetevirens* | 16/12/2021 | Langstone Harbour, UK | L161221SA LD | 50°47'37.71"N | 1° 1'50.91"W | Low | Silt to gravel mudflat sediment type, adjacent seagrass, salt marsh habitats | NGS (13.1) |
| *Cladophora albida* | 16/12/2021 | Langstone Harbour, UK | L161221SA LD | 50°47'37.71"N | 1° 1'50.91"W | Low | Silt to gravel mudflat sediment type, adjacent seagrass, salt marsh habitats | NGS (13.8) |
| *Chaetomorpha linum* | 16/12/2021 | Langstone Harbour, UK | L161221SA LD | 50°47'37.71"N | 1° 1'50.91"W | Low | Silt to gravel mudflat sediment type, adjacent seagrass, salt marsh habitats | NGS (16.5) |
| *Ulva clathrata* | 16/12/2021 | Langstone Harbour, UK | L161221SA LD | 50°47'37.71"N | 1° 1'50.91"W | Low | Silt to gravel mudflat sediment type, adjacent seagrass, salt marsh habitats | NGS (14.4) |
| *Pylaiella littoralis* | 16/12/2021 | Langstone Harbour, UK | L161221SA LD | 50°47'37.71"N | 1° 1'50.91"W | Low | Silt to gravel mudflat sediment type, adjacent seagrass, salt marsh habitats | NGS (39.4) |
| *Cladophora laetevirens* | 16/12/2021 | Langstone Harbour, UK | L161221SA MD | 50°47'38.06"N | 1° 1'51.73"W | Med | Silt to gravel mudflat sediment type, adjacent seagrass, salt marsh habitats | NGS (9.3) |
| *Cladophora albida* | 16/12/2021 | Langstone Harbour, UK | L161221SA MD | 50°47'38.06"N | 1° 1'51.73"W | Med | Silt to gravel mudflat sediment type, adjacent seagrass, salt marsh habitats | NGS (19.4) |
| *Chaetomorpha linum* | 16/12/2021 | Langstone Harbour, UK | L161221SA MD | 50°47'38.06"N | 1° 1'51.73"W | Med | Silt to gravel mudflat sediment type, adjacent seagrass, salt marsh habitats | NGS (20.1) |
| *Chaetomorpha ligustica* | 16/12/2021 | Langstone Harbour, UK | L161221SA MD | 50°47'38.06"N | 1° 1'51.73"W | Med | Silt to gravel mudflat sediment type, adjacent seagrass, salt marsh habitats | NGS (2.5) |
| *Ulva clathrata* | 16/12/2021 | Langstone Harbour, UK | L161221SA MD | 50°47'38.06"N | 1° 1'51.73"W | Med | Silt to gravel mudflat sediment type, adjacent seagrass, salt marsh habitats | NGS (20.6) |
| *Pylaiella littoralis* | 16/12/2021 | Langstone Harbour, UK | L161221SA MD | 50°47'38.06"N | 1° 1'51.73"W | Med | Silt to gravel mudflat sediment type, adjacent seagrass, salt marsh habitats | NGS (27) |
| *Cladophora laetevirens* | 16/12/2021 | Langstone Harbour, UK | L161221SA HD | 50°47'37.66"N | 1° 1'51.25"W | High | Silt to gravel mudflat sediment type, adjacent seagrass, salt marsh habitats | NGS (8.6) |
| *Cladophora albida* | 16/12/2021 | Langstone Harbour, UK | L161221SA HD | 50°47'37.66"N | 1° 1'51.25"W | High | Silt to gravel mudflat sediment type, adjacent seagrass, salt marsh habitats | NGS (13.3) |
| *Chaetomorpha linum* | 16/12/2021 | Langstone Harbour, UK | L161221SA HD | 50°47'37.66"N | 1° 1'51.25"W | High | Silt to gravel mudflat sediment type, adjacent seagrass, salt marsh habitats | NGS (14) |
| *Ulva clathrata* | 16/12/2021 | Langstone Harbour, UK | L161221SA HD | 50°47'37.66"N | 1° 1'51.25"W | High | Silt to gravel mudflat sediment type, adjacent seagrass, salt marsh habitats | NGS (10.9) |
| *Pylaiella littoralis* | 16/12/2021 | Langstone Harbour, UK | L161221SA HD | 50°47'37.66"N | 1° 1'51.25"W | High | Silt to gravel mudflat sediment type, adjacent seagrass, salt marsh habitats | NGS (48.6) |
| *Chaetomorpha ligustica* | 16/12/2021 | Langstone Harbour, UK | L161221SA HD | 50°47'37.66"N | 1° 1'51.25"W | High | Silt to gravel mudflat sediment type, adjacent seagrass, salt marsh habitats | NGS (4.5) |
| *Ulva lacinulata* | 11/03/2021 | Open intertidal estuary, Bay of Veys, France | M2MA_11/03/21 MD | 49°22'19.95"N | 1° 6'9.90"W | Med | Fine sand with silt sediment type, oyster activities at proximity | CAPS(NA) |
| *Ulva sp. LPP* | 11/03/2021 | Open intertidal estuary, Bay of Veys, France | L1MA_11/03/21 LD | 49°22'18.76"N | 1°6'7.84"W | Low | Fine sand with silt sediment type, oyster activities at proximity | CAPS (NA) |
| *Ulva lacinulata* | 08/06/2021 | Open intertidal estuary, Bay of Veys, France | H3JU_08/06/21 HD | 49°22'22.81"N | 1° 6'8.37"W | High | Fine sand with silt sediment type, oyster activities at proximity | CAPS (NA) |
| *Ulva linza* | 08/06/2021 | Open intertidal estuary, Bay of Veys, France | H3JU_08/06/21 HD | 49°22'22.81"N | 1° 6'8.37"W | High | Fine sand with silt sediment type, oyster activities at proximity | CAPS (NA) |
| *Ulva lacinulata* | 08/06/2021 | Open intertidal estuary, Bay of Veys, France | M2JU_08/06/21 MD | 49°22'22.38"N | 1° 6'7.66"W | Med | Fine sand with silt sediment type, oyster activities at proximity | CAPS (NA) |
| *Ulva linza* | 08/06/2021 | Open intertidal estuary, Bay of Veys, France | M2JU_08/06/21 MD | 49°22'22.38"N | 1° 6'7.66"W | Med | Fine sand with silt sediment type, oyster activities at proximity | CAPS (NA) |
| *Ulva lacinulata* | 08/06/2021 | Open intertidal estuary, Bay of Veys, France | L1JU_08/06/21 LD | 49°22'22.31"N | 1° 6'7.53"W | Low | Fine sand with silt sediment type, oyster activities at proximity | CAPS (NA) |
| *Ulva lacinulata* | 09/2021 | Open intertidal estuary, Bay of Veys, France | H3SE_09/21 High | 49°22'24.94"N | 1° 6'6.28"W | High | Fine sand with silt sediment type, oyster activities at proximity | CAPS (NA) |
| *Ulva lacinulata* | 09/2021 | Open intertidal estuary, Bay of Veys, France | M2SE_09/21 Medium | 49°22'25.32"N | 1° 6'7.94"W | Med | Fine sand with silt sediment type, oyster activities at proximity | CAPS (NA) |
| *Ulva australis* | 09/2021 | Open intertidal estuary, Bay of Veys, France | M2SE_09/21 Medium | 49°22'25.32"N | 1° 6'7.94"W | Med | Fine sand with silt sediment type, oyster activities at proximity | CAPS (NA) |
| *Ulva lacinulata* | 09/2021 | Open intertidal estuary, Bay of Veys, France | L1SE_09/21 Low | 49°22'26.00"N | 1° 6'8.48"W | Low | Fine sand with silt sediment type, oyster activities at proximity | CAPS (NA) |
| *Ulva australis* | 09/2021 | Open intertidal estuary, Bay of Veys, France | L1SE_09/21 Low | 49°22'26.00"N | 1° 6'8.48"W | Low | Fine sand with silt sediment type, oyster activities at proximity | CAPS (NA) |
| *Ulva lacinulata* | 14/12/2021 | Open intertidal estuary, Bay of Veys, France | L1DE 14/12/21 LD | 49°22'15.29"N | 1° 6'12.60"W | Low | Fine sand with silt sediment type, oyster activities at proximity | CAPS (NA) |
| *Ulva pseudocurvata* | 14/12/2021 | Open intertidal estuary, Bay of Veys, France | L1DE 14/12/21 LD | 49°22'15.29"N | 1° 6'12.60"W | Low | Fine sand with silt sediment type, oyster activities at proximity | CAPS (NA) |
| *Ulva australis* | 14/12/2021 | Open intertidal estuary, Bay of Veys, France | L1DE 14/12/21 LD | 49°22'15.29"N | 1° 6'12.60"W | Low | Fine sand with silt sediment type, oyster activities at proximity | CAPS (NA) |
| *Ulva lacinulata* | 14/12/2021 | Open intertidal estuary, Bay of Veys, France | L2DE 14/12/21 LD | 49°22'15.19"N | 1° 6'12.98"W | Low | Fine sand with silt sediment type, oyster activities at proximity | CAPS (NA) |
| *Ulva pseudocurvata* | 14/12/2021 | Open intertidal estuary, Bay of Veys, France | L2DE 14/12/21 LD | 49°22'15.19"N | 1° 6'12.98"W | Low | Fine sand with silt sediment type, oyster activities at proximity | CAPS (NA) |
| *Ulva australis* | 14/12/2021 | Open intertidal estuary, Bay of Veys, France | L2DE 14/12/21 LD | 49°22'15.19"N | 1° 6'12.98"W | Low | Fine sand with silt sediment type, oyster activities at proximity | CAPS (NA) |
| *Ulva lacinulata* | 14/12/2021 | Open intertidal estuary, Bay of Veys, France | L3DE 14/12/21 LD | 49°22'15.06"N | 1° 6'13.31"W | Low | Fine sand with silt sediment type, oyster activities at proximity | CAPS (NA) |
| *Ulva pseudocurvata* | 14/12/2021 | Open intertidal estuary, Bay of Veys, France | L3DE 14/12/21 LD | 49°22'15.06"N | 1° 6'13.31"W | Low | Fine sand with silt sediment type, oyster activities at proximity | CAPS (NA) |
| *Ulva australis* | 14/12/2021 | Open intertidal estuary, Bay of Veys, France | L3DE 14/12/21 LD | 49°22'15.06"N | 1° 6'13.31"W | Low | Fine sand with silt sediment type, oyster activities at proximity | CAPS (NA) |
| *Ulva fenestrata* | 14/12/2021 | Open intertidal estuary, Bay of Veys, France | L3DE 14/12/21 LD | 49°22'15.06"N | 1° 6'13.31"W | Low | Fine sand with silt sediment type, oyster activities at proximity | CAPS (NA) |
| *Ulva lacinulata* | 17/09/2021 | Ledano mudflat, Lezardrieux, France | S_LED_2021_9_GEN_D1B_LIB | 48° 27' 43.56"N | 3° 37' 4.22"W | Low | Silt and clay mudflat sediment types with adjacent salt marsh environments | CAPS (NA) |
| *Ulva uncialis* | 17/09/2021 | Ledano mudflat, Lezardrieux, France | S_LED_2021_9_GEN_D1B_LIB | 48° 27' 43.56"N | 3° 37' 4.22"W | Low | Silt and clay mudflat sediment types with adjacent salt marsh environments | CAPS (NA) |
| *Ulva lacinulata* | 17/09/2021 | Ledano mudflat, Lezardrieux, France | S_LED_2021_9_GEN_D2C_LIB | 48° 27' 43.49"N | 3° 37' 4.51"W | Med | Silt and clay mudflat sediment types with adjacent salt marsh environments | CAPS (NA) |
| *Ulva uncialis* | 17/09/2021 | Ledano mudflat, Lezardrieux, France | S_LED_2021_9_GEN_D2C_LIB | 48° 27' 43.49"N | 3° 37' 4.51"W | Med | Silt and clay mudflat sediment types with adjacent salt marsh environments | CAPS (NA) |
| *Ulva lacinulata* | 17/09/2021 | Ledano mudflat, Lezardrieux, France | S_LED_2021_9_GEN_D3C_LIB | 48° 27' 42.84"N | 3° 37' 4.04"W | High | Silt and clay mudflat sediment types with adjacent salt marsh environments | CAPS (NA) |
| *Ulva uncialis* | 17/09/2021 | Ledano mudflat, Lezardrieux, France | S_LED_2021_9_GEN_D3C_LIB | 48° 27' 42.84"N | 3° 37' 4.04"W | High | Silt and clay mudflat sediment types with adjacent salt marsh environments | CAPS (NA) |
| *Ulva lacinulata* | 11/03/2021 | Holes Bay, Poole Harbour, UK | 11/3/21 1HIGH-HB-LM | 50° 43' 10.74"N | 1° 59' 3.95"W | High | Silt and clay sediment types with adjacent salt marsh habitat | NGS (100) |
| *Ulva lacinulata* | 11/03/2021 | Holes Bay, Poole Harbour, UK | 11/3/21 1MED-HB-LM | 50° 43' 6.23"N | 2° 0' 11.61"W | Med | Silt, clay and gravel sediment types with adjacent salt marsh habitat | CAPS (NA) |
| *Ulva lacinulata* | 24/06/2021 | Holes Bay, Poole Harbour, UK | 24/06/21 1HIGH-HB-LM | 50° 43' 11.76"N | 1° 59' 4.05"W | High | Silt and clay sediment types with adjacent salt marsh habitat | NGS (100) |
| *Ulva lacinulata* | 24/06/2021 | Holes Bay, Poole Harbour, UK | 24/06/21 2HIGH-HB-LM | 50° 43' 11.76"N | 1° 59' 4.05"W | High | Silt and clay sediment types with adjacent salt marsh habitat | NGS (95.5) |
| *Ulva compressa* | 24/06/2021 | Holes Bay, Poole Harbour, UK | 24/06/21 2HIGH-HB-LM | 50° 43' 11.76"N | 1° 59' 4.05"W | High | Silt and clay sediment types with adjacent salt marsh habitat | NGS (4.5) |
| *Ulva lacinulata* | 24/06/2021 | Holes Bay, Poole Harbour, UK | 24/06/21 3HIGH-HB-LM | 50° 43' 11.76"N | 1° 59' 4.05"W | High | Silt and clay sediment types with adjacent salt marsh habitat | NGS (100) |
| *Ulva lacinulata* | 24/06/2021 | Holes Bay, Poole Harbour, UK | 24/06/21 3HIGH-HB-EM | 50° 43' 11.76"N | 1° 59' 4.05"W | High | Silt and clay sediment types with adjacent salt marsh habitat | NGS (85.5) |
| *Ulva compressa* | 24/06/2021 | Holes Bay, Poole Harbour, UK | 24/06/21 3HIGH-HB-EM | 50° 43' 11.76"N | 1° 59' 4.05"W | High | Silt and clay sediment types with adjacent salt marsh habitat | NGS (10.6) |
| *Ulva sp. LPP* | 24/06/2021 | Holes Bay, Poole Harbour, UK | 24/06/21 3HIGH-HB-EM | 50° 43' 11.76"N | 1° 59' 4.05"W | High | Silt and clay sediment types with adjacent salt marsh habitat | NGS (3.8) |
| *Ulva lacinulata* | 24/06/2021 | Holes Bay, Poole Harbour, UK | 24/06/21 1MED-HB-LM | 50° 43' 11.73"N | 1° 59' 3.94"W | Med | Silt and clay sediment types with adjacent salt marsh habitat | CAPS (NA) |
| *Ulva lacinulata* | 24/06/2021 | Holes Bay, Poole Harbour, UK | 24/06/21 1MED-HB-LM | 50° 43' 11.73"N | 1° 59' 3.94"W | Med | Silt and clay sediment types with adjacent salt marsh habitat | NGS (100) |
| *Ulva lacinulata* | 25/06/2021 | Holes Bay, Poole Harbour, UK | 25/06/21 1LOW-HB-LM | 50° 43' 8.40"N | 2° 0' 7.51"W | Low | Silt, clay and gravel sediment types with adjacent salt marsh habitat | NGS (91.5) |
| *Ulva sp. LPP* | 25/06/2021 | Holes Bay, Poole Harbour, UK | 25/06/21 1LOW-HB-LM | 50° 43' 8.40"N | 2° 0' 7.51"W | Low | Silt, clay and gravel sediment types with adjacent salt marsh habitat | NGS (8.5) |
| *Ulva lacinulata* | 21/09/2021 | Holes Bay, Poole Harbour, UK | 1 High HB LM 21/09/21 | 50° 43' 13.15"N | 1° 59' 3.80"W | High | Silt and clay sediment types with adjacent salt marsh habitat | CAPS (NA) |
| *Ulva lacinulata* | 21/09/2021 | Holes Bay, Poole Harbour, UK | 1 Med HB LM 21/09/21 | 50° 43' 12.76"N | 1° 59' 3.70"W | Med | Silt and clay sediment types with adjacent salt marsh habitat | CAPS (NA) |
| *Ulva lacinulata* | 22/09/2021 | Holes Bay, Poole Harbour, UK | 1 Low HB LM 22/09/21 | 50° 43' 8.31"N | 2° 0' 7.19"W | Low | Silt, clay and gravel sediment types with adjacent salt marsh habitat | CAPS (NA) |
| *Ulva lacinulata* | 01/12/2021 | Holes Bay, Poole Harbour, UK | 1 Low HB LM 01/12/2021 | 50° 43' 8.31"N | 2° 0' 7.19"W | Low | Silt, clay and gravel sediment types with adjacent salt marsh habitat | CAPS (NA) |
| *Ulva lacinulata* | 02/12/2021 | Holes Bay, Poole Harbour, UK | 2 Med HB LM 02/12/2021 | 50° 43' 6.74"N | 2° 0' 10.50"W | Med | Silt, clay and gravel sediment types with adjacent salt marsh habitat | CAPS (NA) |
| *Ulva lacinulata* | 01/12/2021 | Holes Bay, Poole Harbour, UK | 2 High HB LM 01/12/2021 | 50° 43' 12.14"N | 1° 59' 3.80"W | High | Silt and clay sediment types with adjacent salt marsh habitat | CAPS (NA) |
|  |  |  |  |  |  |  |  |  |

**TABLE S3** Metadata for individual specimens collected during the bulk sampling used for NGS for Holes Bay, UK. See ‘Table S3 Metadata on Individual Specimen Pressings Holes Bay.xlsx’

**TABLE S4** Summary of non-parametric test of analysis of variance using Kruskal-Wallis ANOVA and post-hoc Dunn’s test statistics for within site monthly seaweed biomass data. Summary of non-parametric test of analysis of variance by ranks using Friedman ANOVA and post-hoc Dunn’s test statistics for between site monthly seaweed biomass data. See ‘Table S4 Biomass Statistics.xlsx’

**
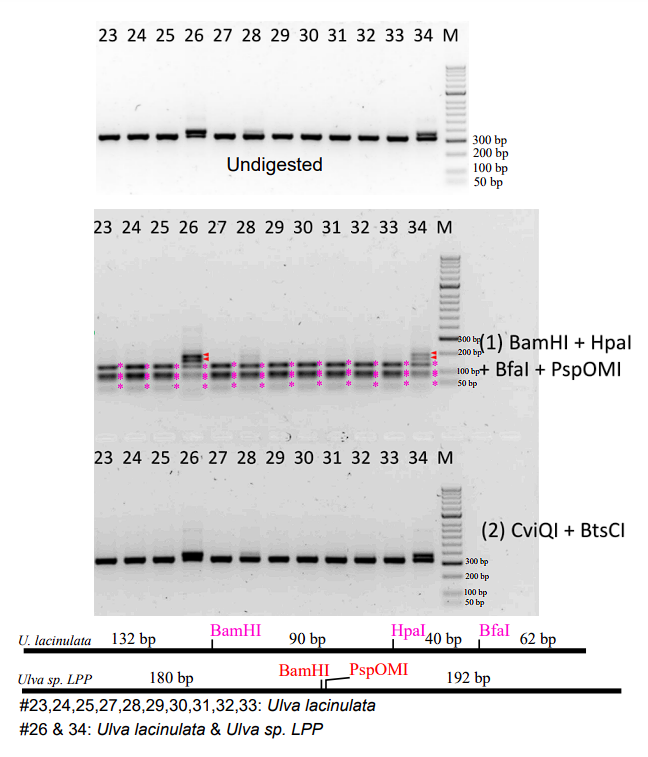
**

**FIGURE S1** DNA band patterns resulting after enzyme digestion of September and December 2021 Holes Bay seaweed samples.


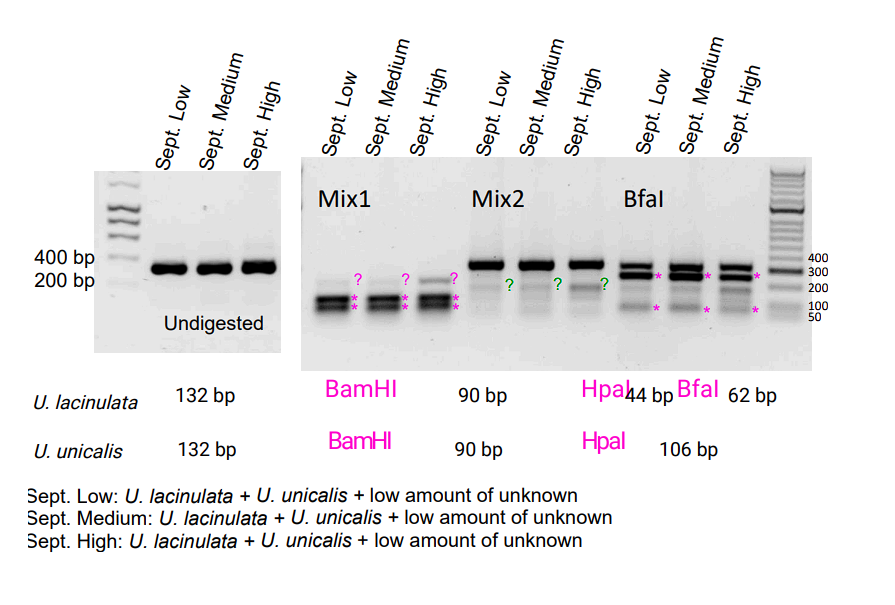


**FIGURE S2** DNA band patterns resulting after enzyme digestion of September 2021 Ledano Estuary seaweed samples.


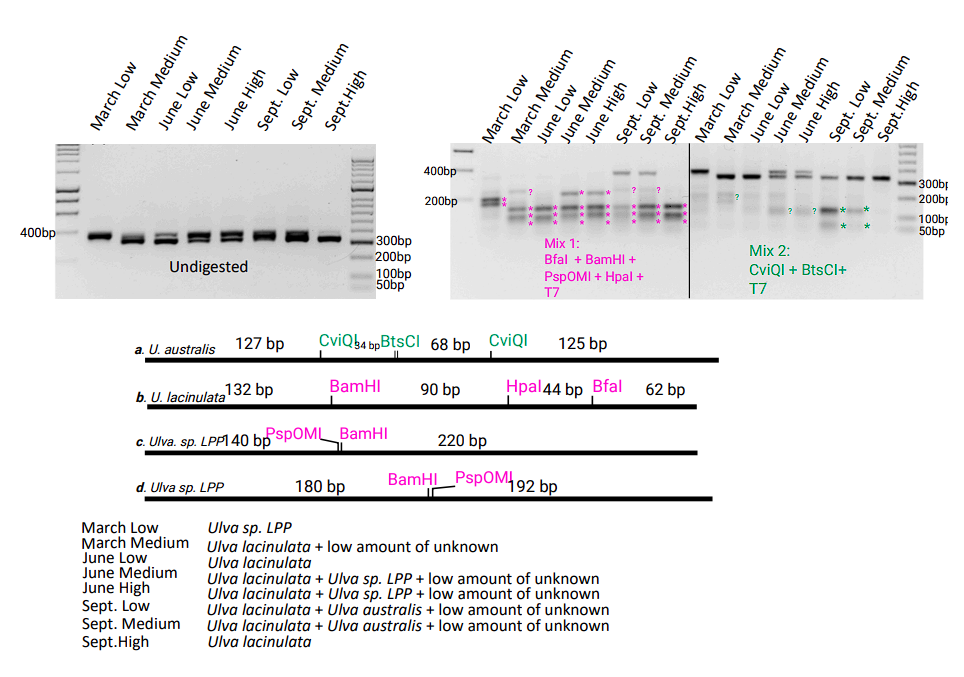


A

B


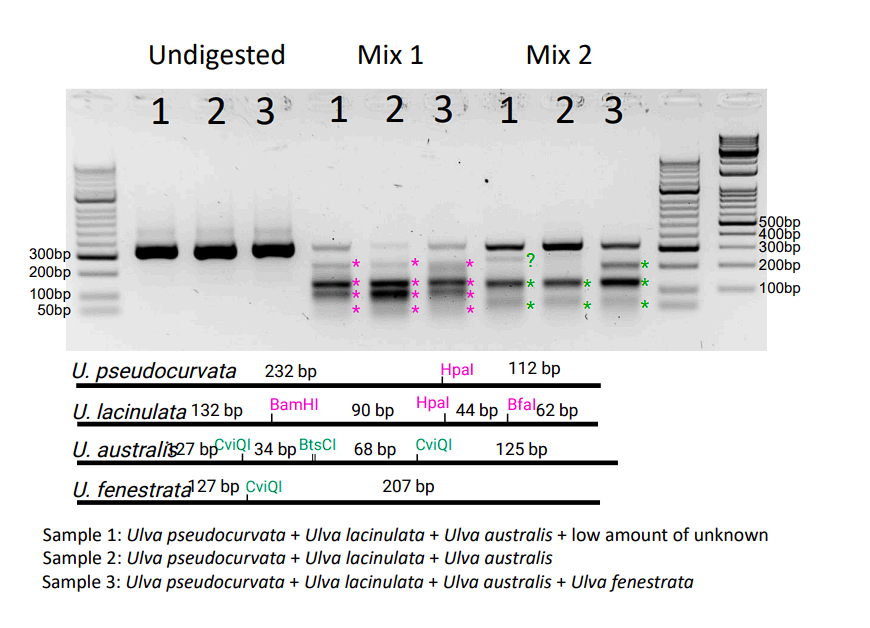


**FIGURE S3** DNA band patterns resulting after enzyme digestion of La Baie des Veys A. March – September 2021 B. December 2021 seaweed samples.
