# Supplementary figures and images for: Long-term prognosis of adults with moderately severe SARS-CoV-2 lower respiratory tract infection managed in primary care: Prospective cohort study
Source: Eur J Gen Pract. 2025 Jun 2;31(1):2501306. doi: 10.1080/13814788.2025.2501306 (PMC12131542; doi:10.1080/13814788.2025.2501306)

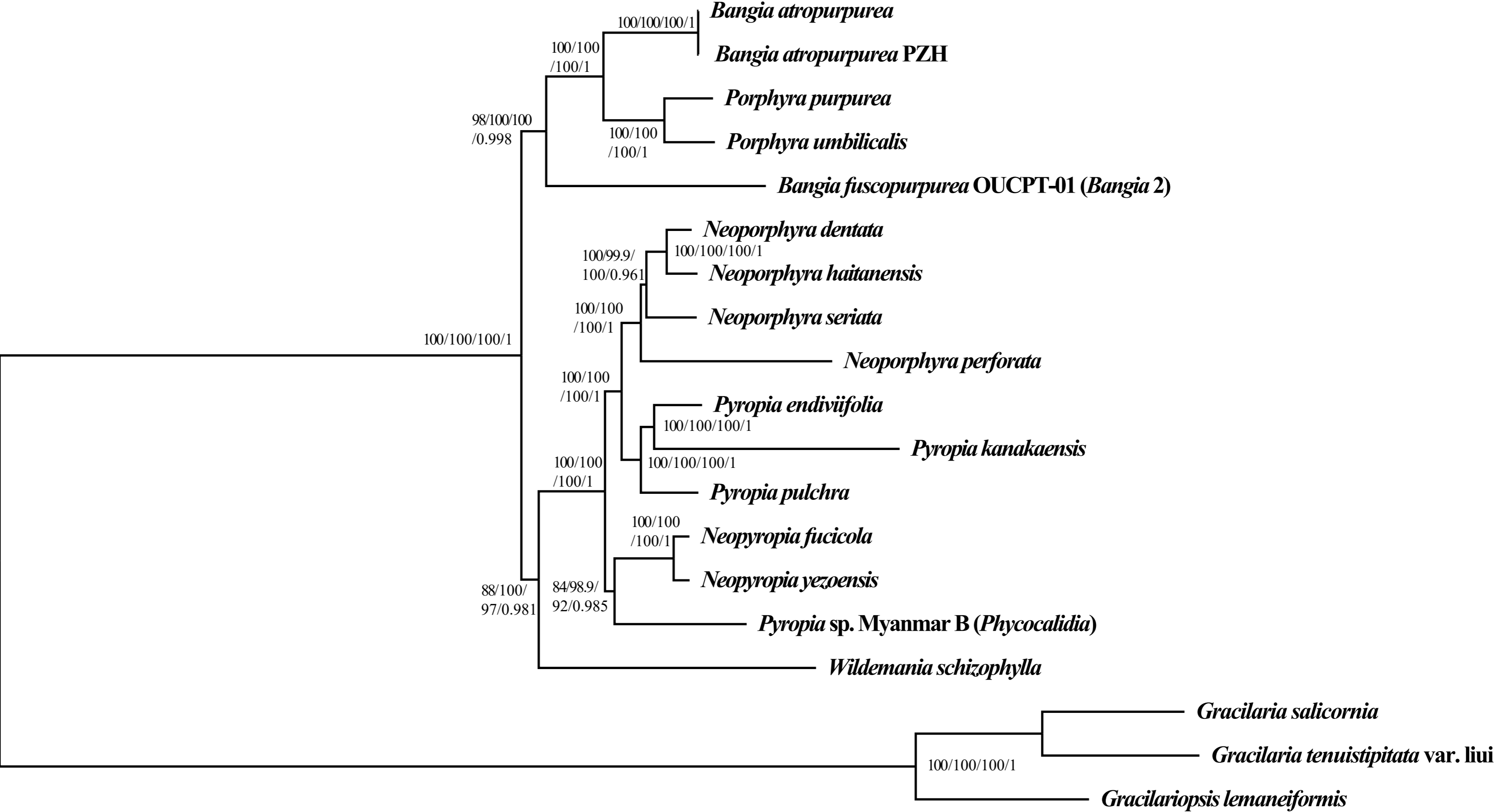

0.2

Supplement: Supplemental Material [file IGEN_A_2501306_SM8918.zip › IGEN_A_2501306_suppl_data/tejp-2024-0108-File005.pdf]

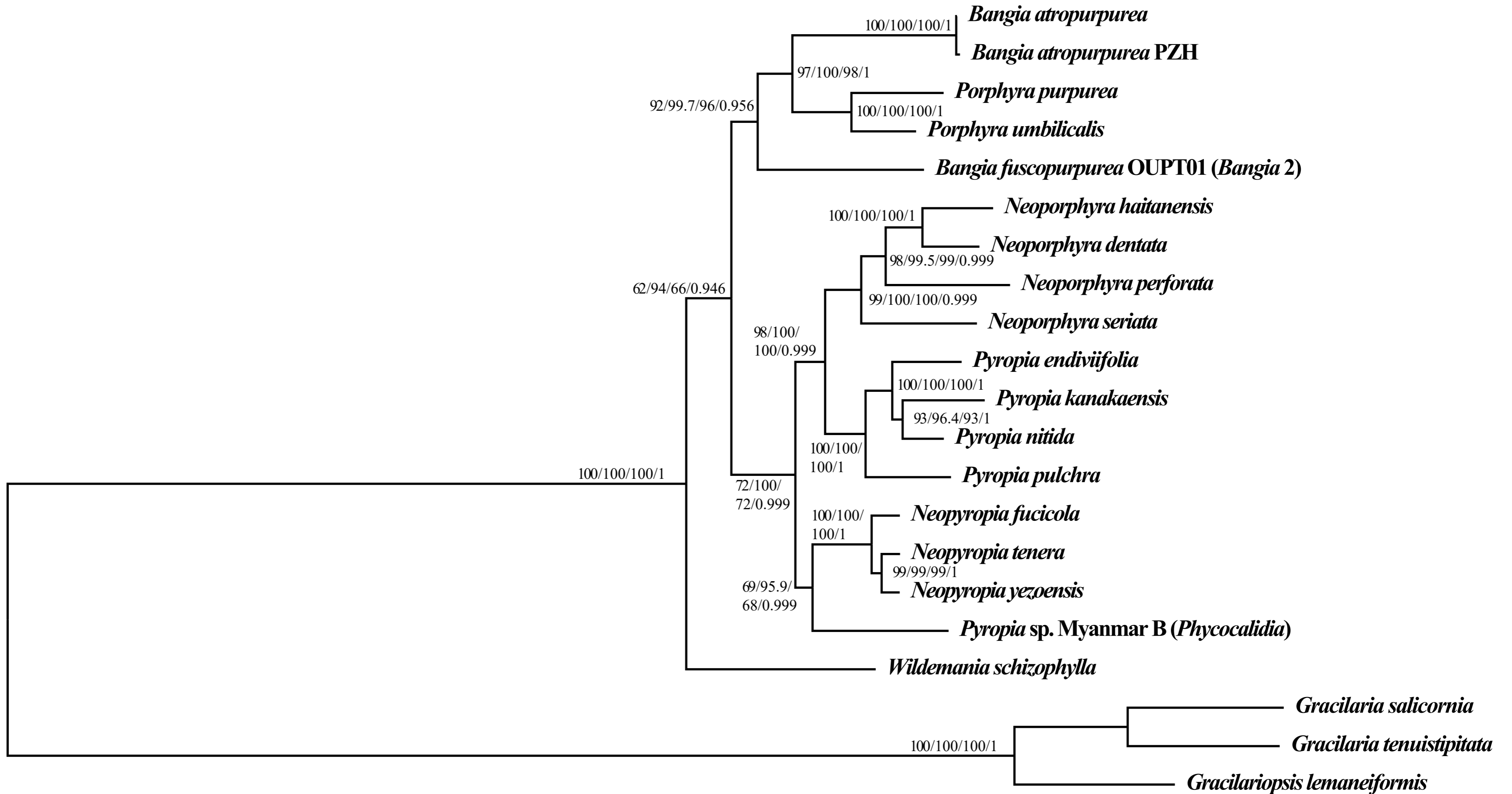

0.1

Supplement: Supplemental Material [file IGEN_A_2501306_SM8918.zip › IGEN_A_2501306_suppl_data/tejp-2024-0108-File006.pdf]

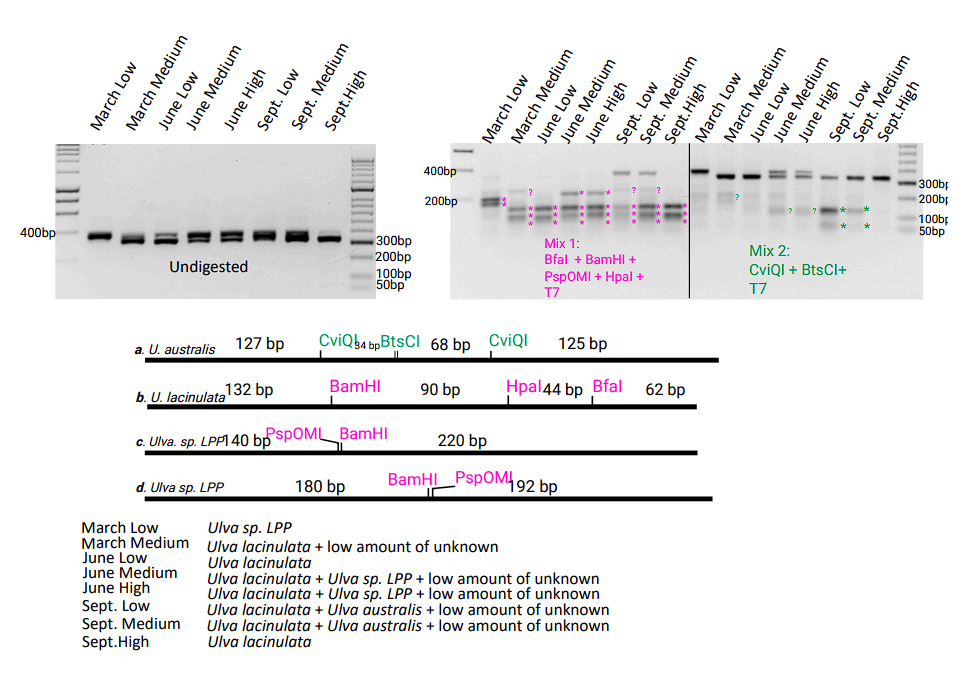

Supplement: Supplemental Material [file IGEN_A_2501306_SM8918.zip › IGEN_A_2501306_suppl_data/tejp-2024-0118-File006.tiff]

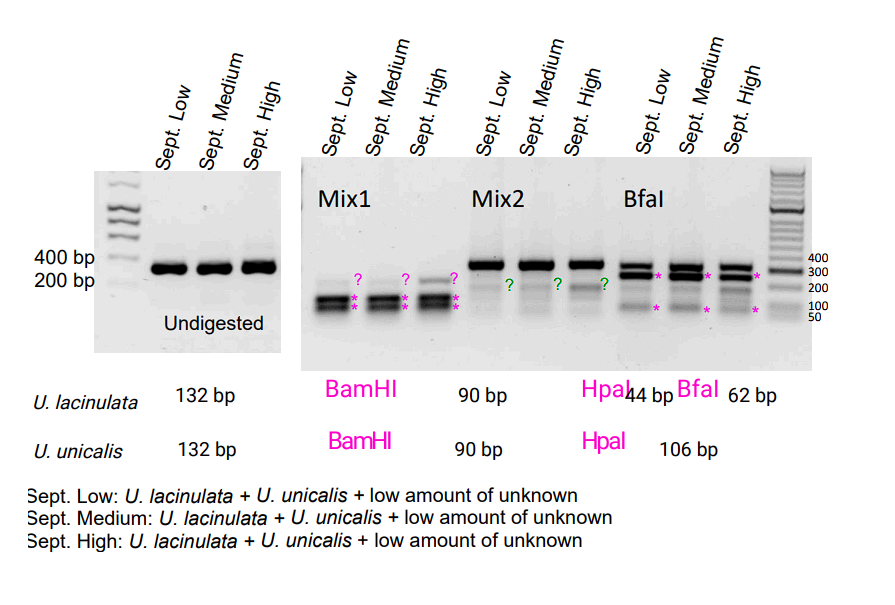

Supplement: Supplemental Material [file IGEN_A_2501306_SM8918.zip › IGEN_A_2501306_suppl_data/tejp-2024-0118-File007.tiff]

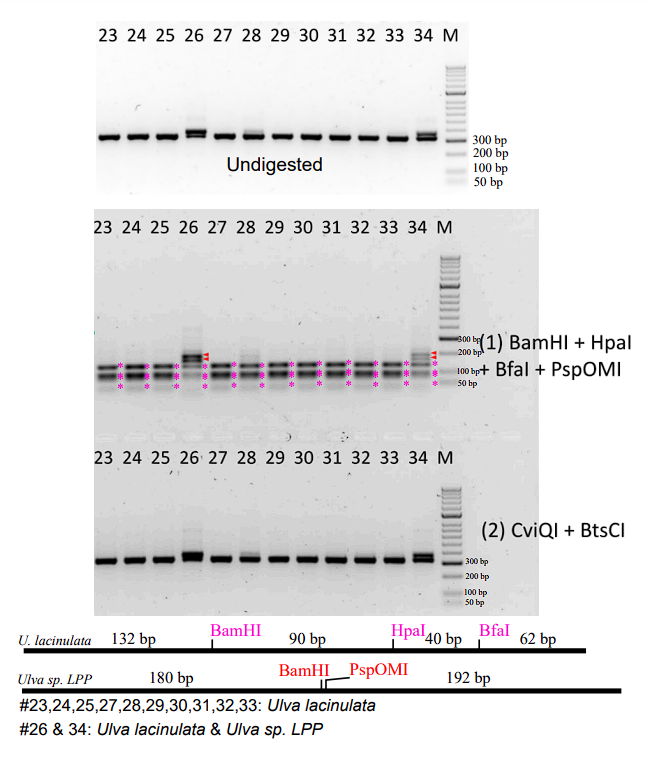

Supplement: Supplemental Material [file IGEN_A_2501306_SM8918.zip › IGEN_A_2501306_suppl_data/tejp-2024-0118-File008.tiff]

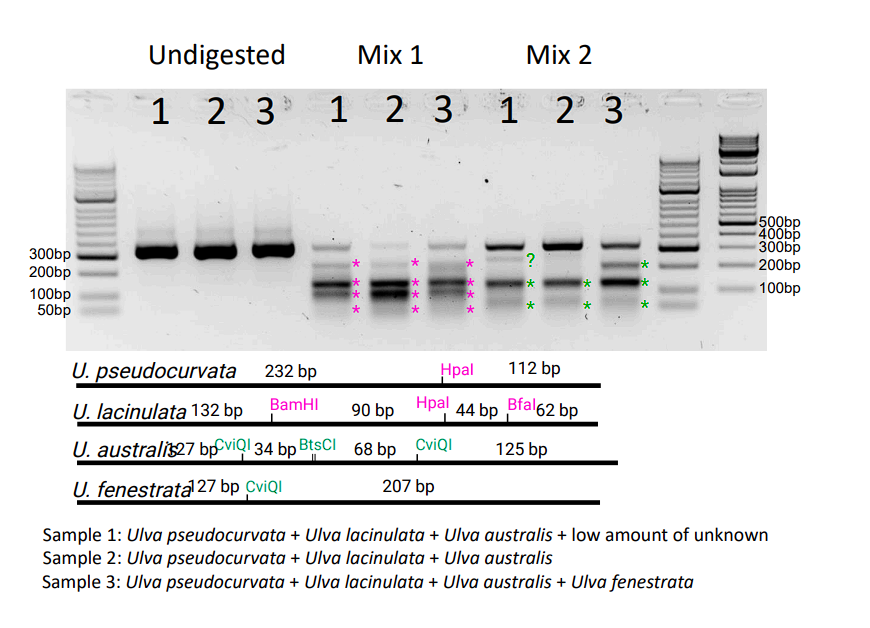

Supplement: Supplemental Material [file IGEN_A_2501306_SM8918.zip › IGEN_A_2501306_suppl_data/tejp-2024-0118-File009.tiff]
